# Study on Multi-Component Modification and Performance Optimization of High-Salt Mine Water Mixed and Sprayed Concrete Based on Response Surface Methodology

**DOI:** 10.3390/ma19183895

**Published:** 2026-09-13

**Authors:** Mao Jing, Kang Peng, Tao Chen

**Affiliations:** School of Resources and Safety Engineering, Central South University, Changsha 410083, China

**Keywords:** mining engineering, mine water, response surface methodology, shotcrete, corrosion resistance

## Abstract

The deep-sea tunnels at the Sanshan Island Gold Mine are subjected to extreme conditions characterized by high stress and complex erosion resulting from high mineralization. Under these conditions, conventional shotcrete is prone to performance degradation and insufficient durability, posing a threat to the long-term safety of the tunnels. At the same time, mine water is difficult to recycle on-site. To address these engineering challenges, this study utilized fly ash (FA), S105-grade ground granulated blast furnace slag (GGBS), polypropylene coarse fiber (PPCF), and hydroxypropyl methylcellulose (HPMC) as modifying components and employed the response surface method (RSM) to optimize the mix design of mine water-blended shotcrete. The study selected compressive strength, direct shear strength, and chloride ion electrical flux at 6 h as response indicators and constructed a quadratic polynomial regression model. Analysis of variance and goodness-of-fit tests indicated that the model possessed good significance and reliability of fit. Based on this model, the optimal mix design was determined: an FA/GGBS blend ratio of 3:7, a cement replacement rate of 20%, a PPCF content of 3.3%, and an HPMC content of 0.18%. Performance testing showed that the optimal mixture achieved a compressive strength of 25.24 MPa, a direct shear strength of 8.08 MPa, and a chloride ion electrical flux of 778 C after 6 h. Compared to the control group, its peak compressive strength decreased by only 9.98%, while its residual strength increased significantly; direct shear strength increased by 18.1%, and electrical flux decreased by 33.8%. This indicates that the material’s mechanical load-bearing capacity, deformation coordination, and corrosion resistance have been enhanced in a synergistic manner. Field industrial trials have verified that this modified concrete possesses excellent ductile yield characteristics, can effectively suppress water seepage in mine tunnels, is capable of withstanding extreme underground operating conditions, and enables the efficient reuse of mine water resources.

## 1. Introduction

**Figure 1:**
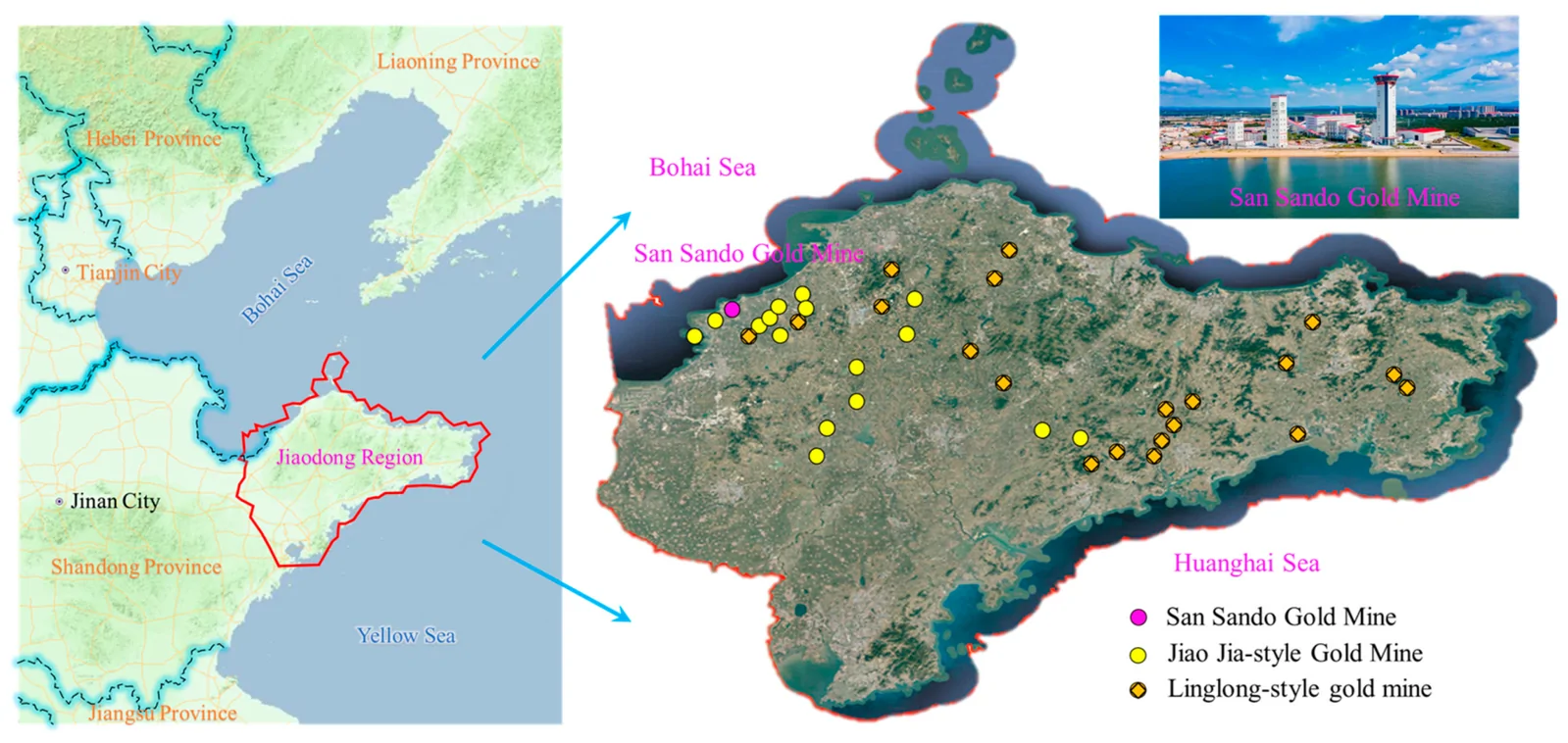
Distribution of gold deposits in the Jiaodong–Bohai Bay area.

As the trend toward deep mining intensifies globally, deep mining operations commonly face complex operating conditions such as high in situ stress, severe erosion, and high osmotic pressure [1,2], posing severe challenges to the load-bearing capacity, durability, and long-term performance of tunnel support systems. Among these factors, high-salinity mine water—rich in corrosive media such as Cl^−^, SO_4_^2−^, and Mg^2+^—is the primary factor contributing to the degradation of shotcrete support materials [3,4]. Taking the Sanshan Island Gold Mine on the Jiaodong Peninsula as an example, the mining area is bordered by the sea on three sides and features widespread Quaternary loose aquifers, making it significantly susceptible to seawater intrusion (Figure 1); Peng Kang and others have conducted extensive research on mining safety issues at this deposit [5,6]. Their studies indicate that the Sanshan Island Gold Mine is bordered by the sea on three sides, and extensive Quaternary loose aquifers are widely developed. Due to seawater intrusion, the fissure water underground is enriched with corrosive ions such as Cl^−^, SO_4_^2−^, and Mg^2+^, and the ion concentrations vary significantly depending on the sampling level and location; chloride ion concentrations in deep fissure water samples generally reach 20,000 mg/L or higher. Once these ions penetrate the shotcrete, they damage the hydration structure of the cement paste, triggering a series of degradation issues such as surface spalling, cracking and flaking, and a decline in mechanical strength, which significantly undermine the stability of the support system and shorten its service life [7,8,9]. It is worth noting that the use of mine water to prepare shotcrete in this mining area enables the resource utilization of mine water, reduces transportation energy consumption and costs, and facilitates underground construction, aligning with the concept of a green mine. However, the current shotcrete mixture in the mining area consists solely of a basic blend of “mine water + cement + river sand.” The resulting hydrated structure is porous, and when combined with high-salt-ion corrosion, the risk of support failure is significant. There is an urgent need to develop new support materials suited to the region’s high-salt environment to address this degradation issue.

Shotcrete is a core technology for tunnel support; its mechanical properties and resistance to erosion directly determine the effectiveness and long-term stability of deep-level support systems [10,11]. However, traditional shotcrete systems have inherent limitations in high-salt environments. To address this challenge, researchers both domestically and internationally have conducted extensive studies in areas such as modification with mineral admixtures, fiber reinforcement, and the utilization of unconventional water resources, achieving phased progress.

In the field of mechanical properties of fiber-reinforced concrete, existing studies [12,13] have confirmed that adding structural fibers to cement-based composites can significantly enhance their tensile, shear, crack resistance, and impact resistance properties. Currently, the fiber-reinforced concrete commonly used in engineering applications includes steel fibers, glass fibers, carbon fibers, and organic synthetic fibers [14,15]; however, each type of fiber has distinct limitations: steel fibers are prone to corrosion, carbon fibers have low shear strength, and glass fibers exhibit poor dispersion in concrete [16,17,18]. In contrast, polypropylene fibers are more suitable for sprayed concrete tunnel support due to their low cost, high corrosion resistance, excellent stability, and good dispersion. Further research indicates that the dosage of polypropylene coarse fibers (PPCF), based on the total mass of the concrete, should be controlled within the range of 3 wt% to 7 wt%. An appropriate dosage can enhance the concrete’s ultimate load-bearing capacity and deformation toughness, whereas an excessive dosage will weaken the load-bearing performance of the concrete matrix [19].

Improving water resistance is a key factor in optimizing the performance of shotcrete [20], and mineral admixtures (such as industrial waste residues) are an effective means of achieving this goal—they not only enhance the durability of concrete but also reduce cement consumption, lower costs, and protect the environment. Fly ash (FA) and granulated blast furnace slag powder (GGBS), as commonly used mineral admixtures, are frequently employed as cement substitutes to improve the microstructure of the concrete interface transition zone and enhance corrosion resistance. However, their drawbacks are equally evident: replacing cement inhibits early-stage cement hydration activity, thereby weakening the mechanical properties of concrete [21]; moreover, as the replacement ratio (10–50%) increases, the decline in mechanical properties becomes increasingly pronounced, with only impermeability showing a marked improvement as the replacement ratio rises. It is worth noting that the combined use of FA and GGBS can compensate for the shortcomings of using either material alone: GGBS has high reactivity, and when used together with FA to replace cement, it produces a “synergistic effect” that both reduces the content of hydrated calcium hydroxide and improves the density of the matrix [22]. Studies have confirmed that when the composite admixture (GGBS + FA) replaces 20% of the cement, it ensures good impermeability of the material with virtually no impact on the mechanical properties of the concrete [23]. Furthermore, hydroxypropyl methylcellulose ether (HPMC), acting as a thickening agent, can reduce bubble aggregation and adhesion by adsorbing air, resulting in a more uniform distribution of bubbles and pores in the hardened concrete; its excellent salt resistance, water retention, and thixotropic thickening properties can specifically address challenges such as concrete degradation, cracking, and high construction rebound in the highly corrosive, high-temperature, and complex surrounding rock environments of underground gold mines, ultimately significantly improving the durability of support structures and construction efficiency [24,25,26].

Although the existing research has yielded the aforementioned insights, it is difficult to directly apply these conclusions to the unique operating conditions at the Sanshan Island Gold Mine—characterized by deep-seated high stress, combined high-salinity corrosion, and mine water mixing—and a significant gap still exists between theoretical findings and engineering practice. Specifically, existing research has two main limitations: First, in terms of component combinations, most existing studies on fiber-reinforced modification focus on single-additive PPCF or mixed modification using PPCF combined with steel fibers and carbon fibers. There has been a lack of systematic investigation into the mixing ratio coupling patterns for the “FA–GGBS dual-additive + PPCF + HPMC + mine water” multi-component synergistic modification system. Second, most laboratory tests have been conducted using freshwater mixing and standard curing conditions; there are few reports on the performance of multi-component modified concrete using high-salinity mine water as the mixing medium.

Therefore, guided by the support requirements for deep tunnels at the Sanshan Island Gold Mine and taking into account the ionic characteristics of mine water, this study selected the FA-GGBS composite admixture system and used PPCF and HPMC as modifying components to prepare a multi-component modified shotcrete suitable for mixing with mine water. By establishing a multi-factor synergistic mixing ratio system for multi-component modified shotcrete comprising “FA-GGBS–PPCF–HPMC–mine water,” this study moves away from the traditional “cement + river sand + fresh water” base mix; The Response Surface Methodology (RSM) was systematically applied to elucidate the influence patterns of three factors—the FA-GGBS composite blending ratio, PPCF dosage, and HPMC dosage—on the concrete’s compressive strength, shear strength, and chloride ion electrical conductivity, and a multi-objective regression prediction model was established. The mechanism of multi-component synergistic modification was elucidated based on macroscopic mechanical properties and impermeability. Furthermore, field application tests at the Sanshan Island Gold Mine were conducted to verify the engineering suitability and in-service performance of the new shotcrete, thereby providing both theoretical and practical support for the upgrading of support materials in deep mine tunnels.

## 2. Materials and Methods

### 2.1. Materials and Specimen Preparation

The shotcrete in this study was prepared from PO 42.5 ordinary Portland cement, S105-grade ground granulated blast-furnace slag (GGBS, Henan Borun Casting Materials Co., Ltd., Zhengzhou, China), Class I fly ash (FA, Gongyi Borun Refractory Materials Co., Ltd., Gongyi, China), HPMC (Jinzhou Fuqiang Fine Chemical Co., Ltd., Jinzhou, China), and polypropylene coarse fiber, batched at the specified mix ratios. S105 GGBS and Class I FA are both reactive mineral admixtures widely used in concrete engineering (Table 1). The HPMC is a non-ionic cellulose ether with a viscosity of 200,000 mPa.s, acting mainly to retain water, thicken the mix, and improve workability. PPCF disperses well in the cement matrix and bonds favorably with it, while offering high tensile strength, strong acid–alkali and rust resistance, and safe handling. The PPCF used measured 30 mm in length and 0.8 mm in diameter; its key properties are listed in Table 2 (elastic modulus ≥ 3.5 GPa, elongation at break ≥ 15%, density 0.91 g/cm^3^). For the aggregate system, medium-grade sand (yellow sand) with a particle size of 2–5 mm was used, as referenced from the underground concrete preparation operations at the Sanshandao Gold Mine (Table 2).

The mixing water used in the experiment was prepared according to the actual sampling data of the fissure water system in the Sanshandao Gold Mine. On the basis of the test and analysis results of the actual mine water sample, four reagents, namely sodium chloride, crystalline sodium sulfate, anhydrous calcium chloride and magnesium chloride hexahydrate, were dissolved stepwise in deionized water at the corresponding ion concentration ratios to prepare the simulated mine water [27], and the detailed proportioning parameters are presented in Table 3.

The preparation scheme of concrete specimens was carried out with reference to the actual construction scheme of shotcrete in the underground of Sanshandao Gold Mine. The benchmark mix proportion was set as follows: sand–cement ratio (S/C) = 3.5; water–cement ratio (W/C) = 0.42. The specimens were divided into two groups: the ordinary shotcrete group and the test shotcrete group. The ordinary shotcrete was prepared only with cement, river sand and mine water according to the benchmark mix proportion; on the basis of ordinary shotcrete, the test shotcrete was mixed with GGBS, FA, HPMC and PPCF. The specific preparation process is as follows.

**Figure 2:**
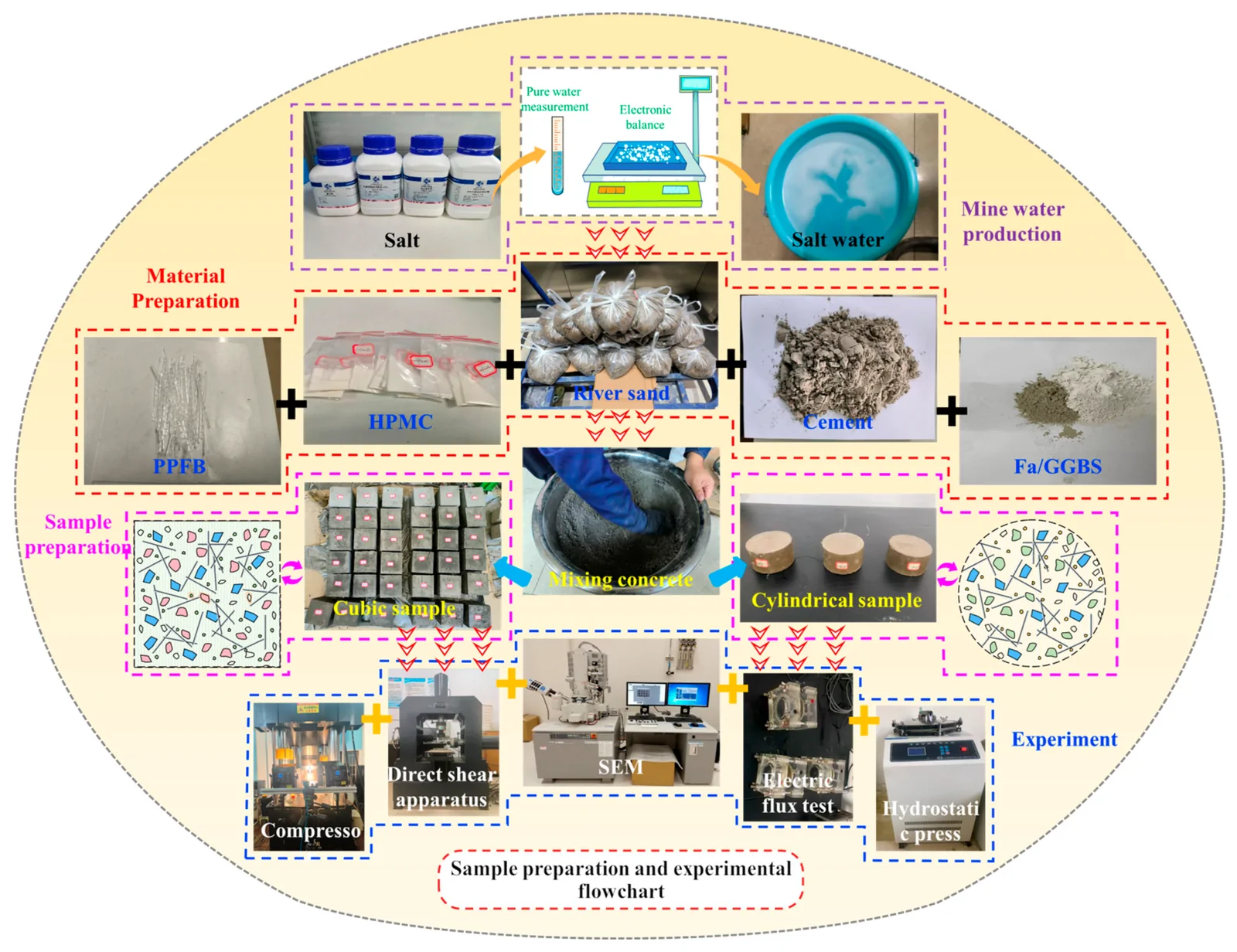
Sample preparation and test flowchart (Solid arrows indicate the sequential procedure of material preparation, concrete mixing, specimen fabrication and mechanical testing; plus signs represent the mixing of different raw materials; circular arrows refer to the schematic illustration of the internal microstructure of the prepared specimens).

First, all components of raw materials were accurately weighed according to the experimental scheme (ordinary group: cement, river sand; test group: cement, river sand, GGBS, FA, HPMC, PPCF). Meanwhile, simulated mine water was prepared in the same way as mixing water with reference to the above method. Subsequently, GGBS, FA, HPMC, PPCF, cement and river sand were fed into the mixer in sequence, followed by dry mixing for 120 s until all materials were uniformly mixed without agglomeration or caking. Then, the preset proportion of mixing water was added to the uniformly dry-mixed materials, and stirring was continued until the workability of the mixture was uniform. After that, the mixture was poured into 100 mm × 100 mm × 100 mm triple rigid plastic molds and Φ100 mm × 50 mm cylindrical plastic molds, respectively, and placed on a shaking table for compaction until no obvious bubbles overflowed from the surface of the mixture in the molds. After vibration compaction, the molds were left to stand for curing for 24 h before demolding. Finally, the two groups of demolded specimens were uniformly transferred into a standard constant-temperature and -humidity curing chamber, where the curing parameters were controlled to be consistent with the standing environment (temperature: 20 ± 2 °C, relative humidity: ≥95%). Standard curing was carried out, and the specimens were taken out for standby after curing to 28-day age, which were used for subsequent relevant mechanical performance and durability tests. The process flow of material preparation and performance testing is shown in Figure 2.

### 2.2. Mechanical Property Tests

Mechanical property tests were carried out using an INSTRON-1346 electro-hydraulic servo universal testing machine (Instron Corporation, High Wycombe, UK) at the Advanced Research Institute of Central South University. The machine has a maximum axial load of 2000 kN, a maximum lateral load of 250 kN, a load control accuracy of ≤±0.5%, a piston stroke of ±150 mm, and a load measurement accuracy of ±0.02% FS [28,29]. The loading mode was displacement control at a rate of 0.1 mm/min. The compressive strength of the concrete specimens is

(1) $$f_{cu}=0.95\frac{F}{A}$$

Direct shear strength was measured using a multifunctional rock direct shear apparatus. L-shaped cushion blocks with a thickness of 2 cm and a width of 10 cm were arranged on the bottom and top surfaces of each cubic specimen, respectively. A pre-pressure of 25 kN was applied to the prestressed screw to ensure failure at the joint surface of the specimen and eliminate inelastic deformation. Loading was applied at a rate of 5 kN/min until the specimen failed or lost bearing capacity. Cubic shotcrete specimens with dimensions of 100 mm × 100 mm were used in the direct shear tests. Note: The original Chinese text had an incomplete sentence for compressive strength formula/expression; if you provide the complete formula, it can be translated and typeset in standard academic format.

### 2.3. Test Method for Chloride Ion Resistance

The chloride ion penetration resistance of concrete was determined via the electric flux method using a multifunctional concrete durability comprehensive tester. Cylindrical specimens with dimensions of Φ100 mm × 50 mm were cast for this test. Before the test, specimens were ground, polished and cleaned, followed by vacuum saturation. The saturated specimens were installed in a test cell, where the anode and cathode compartments were filled with 0.3 mol/L NaOH solution and 3 wt% NaCl solution, respectively. After confirming satisfactory sealing, a constant DC voltage of 60 V was applied axially across the specimen. The total electric charge passing through the specimen within 6 h was automatically recorded to determine the electric flux of the specimen.

### 2.4. RSM

**Figure 3:**
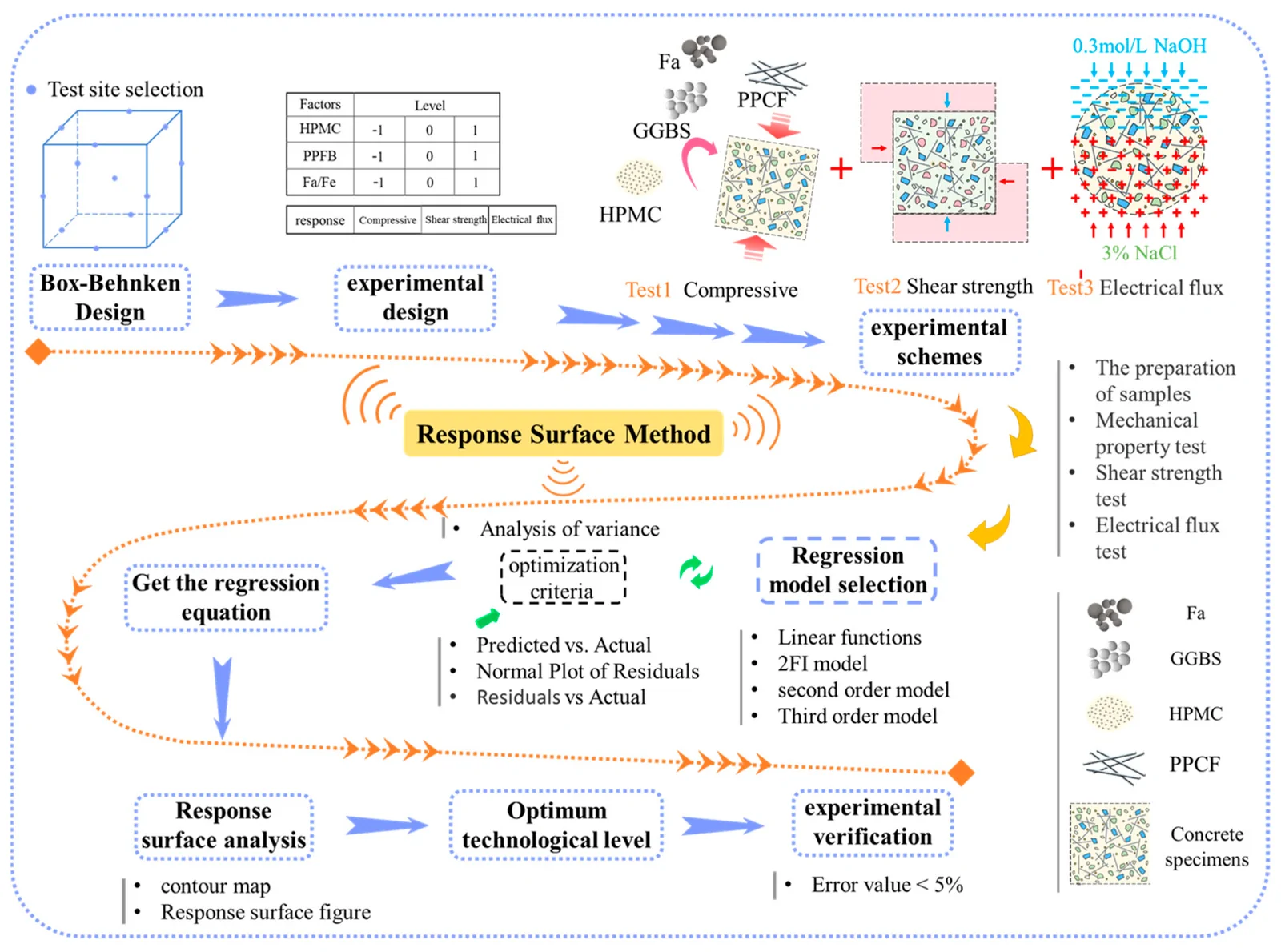
Flow chart of experimental design based on response surface methodology [31].

Common RSM designs include full factorial design (FFD), Box–Behnken design (BBD), and central composite design (CCD). FFD demands many runs yet gives lower model accuracy; CCD and BBD are preferred for three-factor studies because of their higher accuracy. Relative to CCD, BBD avoids axial points, needs fewer runs, and keeps factors within a smaller range [30]. This study therefore adopted BBD to build the response-surface model (Figure 3). RSM relates factors to responses through five steps: data collection, model building, model verification, parameter optimization, and validation. The polynomial response Y is expressed as [31]

(2) $$Y=\beta_{0}+\sum_{i=1}^{K}\beta_{i}X_{i}+\sum_{i=1}^{K}\beta_{ii}X_{i}^{2}+\sum_{i=1}^{K-1}\sum_{j=1}^{K}\beta_{ij}X_{i}X_{j}+\varepsilon$$

Here, Y is the response value of the concrete specimen; Xi and Xj are independent variables (i, j = 1, 2, 3, …, k); βi and βii are the coefficients of the linear terms, and βij is the coefficient of the quadratic term; ε represents the error; and K is a variable. ANOVA was used to fit the model. Significance tests were run on the linear, two-factor-interaction (2FI), quadratic, and cubic models; the overall model and each term were judged by *p*-value. Residual-versus-predicted and predicted-versus-actual plots checked model adequacy and selected the best fit. Response-surface and contour plots then visualized how each factor (fiber, HPMC, FA/GGBS) affected the responses. Finally, optimization targets for the admixture contents were set, and the optimal parameters solved and validated experimentally. The RSM workflow is shown in Figure 3.

### 2.5. SEM and EDS Tests

A Quanta 250 scanning electron microscope manufactured by FEI (USA) was used to observe the micro-morphology of the samples, and a supporting EDS analyzer was employed for qualitative and quantitative analysis of elements in micro-regions, providing support for the study on the structure–composition correlation of the sealing material. SEM images were collected in low-vacuum mode, and EDS was synchronously linked with SEM for fixed-point scanning. The core parameters were set as follows: accelerating voltage of 0.2–30 kV, effective detection area of the EDX detector chip of 10 mm, energy resolution > 129 eV, EDS detection range of B-U, and SEM magnification of 10–100,000 times. Appropriate samples were selected and fixed with conductive adhesive, sputter-coated with a thin gold layer, then placed on the sample stage and evacuated. The SEM parameters were adjusted to collect micro-images of multiple regions, and then EDS scanning was initiated in typical regions to sort out the detection data.

## 3. Results and Analysis

### 3.1. Mix Proportion Optimization of Materials Based on RSM

#### 3.1.1. Experimental Design of RSM

Using the Design-Export experimental design software, a three-factor, three-level design was conducted. This resulted in an L17(3^3^) design table, with a total of 17 test groups. Specifically, the HPMC content ranged from a minimum of 0.1% to a maximum of 0.6%; the polypropylene coarse fiber content ranged from a minimum of 3% to a maximum of 7%; and the FA/GGBS powder ratio ranged from a minimum of 3/7 to a maximum of 7/3. The corresponding levels for each design factor are shown in Table 4.

#### 3.1.2. Model Establishment and Validation

To further explore the intrinsic correlation between each influencing factor and the mechanical and durability properties of shotcrete used in gold mine roadways, a regression fitting analysis was performed on the experimental data in Table 5 with Design-Expert 10 software. Nonlinear fitting comparisons among different models were conducted using the sum of squared deviations from the mean, fitting error and statistical test results as judgment criteria, and the results demonstrated that the quadratic regression model achieved the optimal fitting accuracy. Accordingly, two-factor quadratic regression equations were adopted to establish surface fitting models for 28 d compressive strength, direct shear strength and chloride ion electric flux, with the corresponding regression equations denoted as Y_1_, Y_2_ and Y_3_.

Y_1_ = 23.46 − 2.087X_1_−1.77X_2_ −1.35X_3_ + 0.0200X_1_X_3_ + 0.0125X_1_X_2_ + 0.1125X_2_X_3_-X_1_^2^ −0.901X_2_^2^ −1085X_3_^2^ (3)

Y_2_ = 7.92 − 0.1300X_1_ − 0.0662X_2_ − 0.4538X_3_ + 0.055X_1_X_3_ − 0.0025X_2_X_3_ − 0.3013X_1_^2^ − 0.6638X_2_^2^ − 0.0437X_3_^2^ (4)

Y_3_ = 885.4 − 9.00X_1_ + 61.75X_2_ + 80.75X_3_− 0.25X_1_X_2_ + 2.75X_1_X_3_ + 7.25X_2_X_3_ + 39.68X_1_^2^ + 35.18X_2_^2^ − 15.33X_3_^2^ (5)

ANOVA allows for the acquisition of the *p*-value for each coefficient in the model, which is widely recognized as a core tool for verifying model significance [32]. As shown in Table 6, the *p*-values of the significance tests for all three regression models are less than 0.0001, indicating that the established regression models are statistically significant at a high level. The *p*-values of HPMC content, fiber content and FA/GGBS ratio in all three regression models are all less than 0.05, suggesting that HPMC content, fiber content and FA/GGBS ratio exert significant interactive effects in each model. In addition, the *p*-values of the lack-of-fit terms for the three models are all greater than 0.05, implying that the lack-of-fit of the models is not significant and the fitting effect is reliable. Based on the above test results, the models can satisfactorily fit the experimental data [33].

Table 7 presents the reliability test results of the regression models. The goodness of fit of the models is mainly evaluated by the consistency between the coefficient of determination (R2) and the adjusted coefficient of determination (Adjusted R^2^). The reliability should meet the following criteria: coefficient of variation (C.V.) < 10%, adequate precision > 4, and the difference between Adjusted R^2^ and predicted coefficient of determination (Predicted R^2^) < 0.2 [34]. As can be seen from Table 6, the R^2^ values of the models for compressive strength, direct shear strength and chloride ion electric flux are 0.9989, 0.9857 and 0.9936, respectively, all approaching 1, which indicates an extremely high fitting degree. Further data reveal that the differences between Adjusted R^2^ and Predicted R^2^ for the three models are 0.0071, 0.0253 and 0.0506, respectively, all far below the critical threshold. Meanwhile, the C.V. values are 0.3838%, 0.8268% and 0.7718%, and the adequate precision values reach as high as 94.2792, 32.1686 and 43.2427, all satisfying the evaluation criteria. This confirms that the established regression models can be adopted for the subsequent analysis of the influence laws of various factors on the performance of shotcrete applied in gold mine roadways.

**Figure 4:**
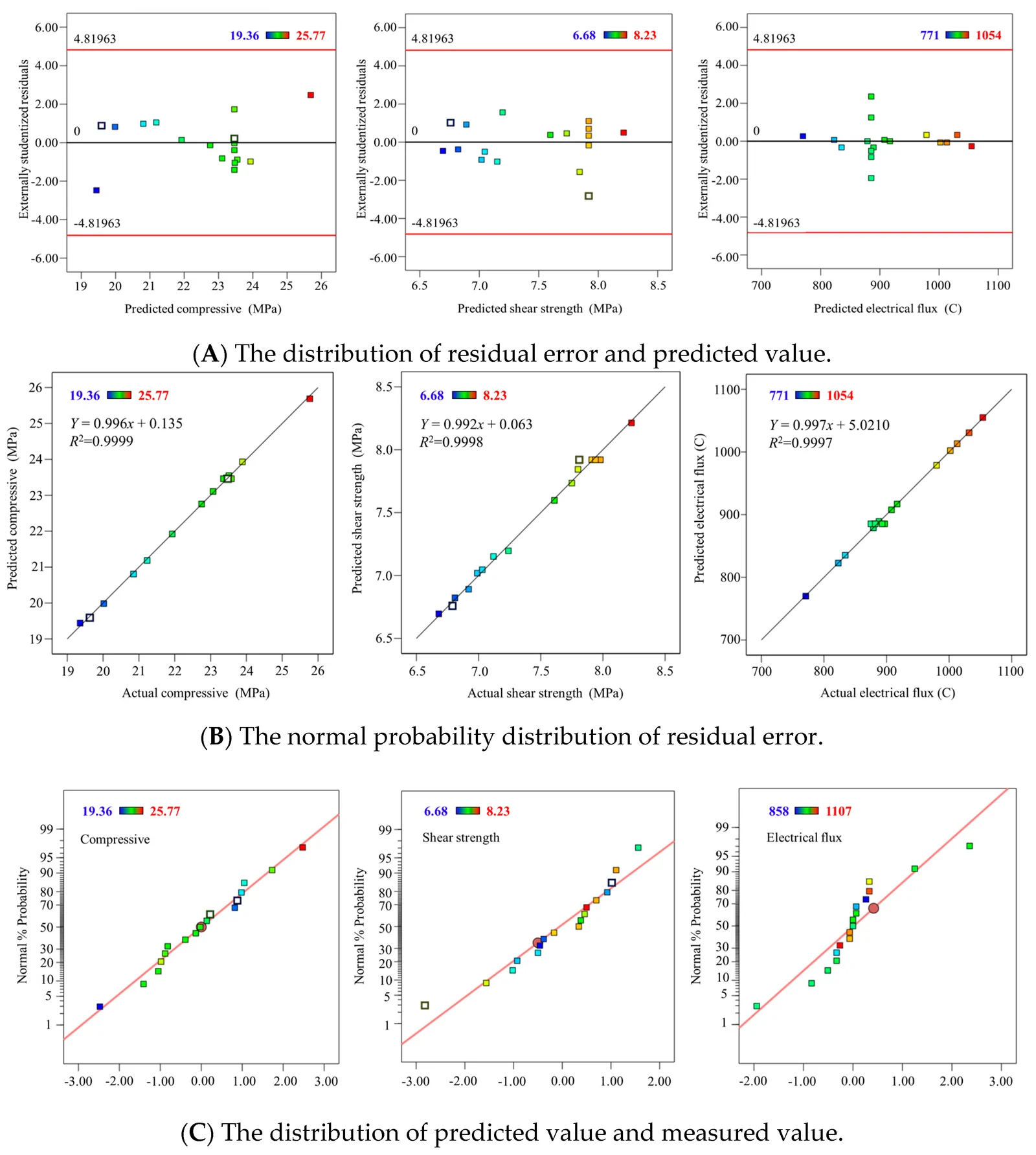
(**A**–**C**) show the distribution of residual error and predicted value, the normal probability distribution of residual error and the distribution of predicted value and measured value of compressive strength respectively.

Besides the above methods, Pareto charts, residual analysis and correlation analysis between predicted and experimental values are also important means to verify model validity [35]. Figure 4 shows the residual-predicted value scatter plots of the regression models with three factors: HPMC content, fiber content and FA/GGBS composite ratio. As observed in Figure 4A, all residual points are randomly scattered without aggregation or trend, verifying the excellent adaptability of the models to the experimental data. Figure 4B displays the residual distribution of the three-factor regression models, where the residual points are uniformly distributed on both sides of the zero line without regular trends or outliers, demonstrating that the residuals conform to the normal distribution and the models can effectively eliminate random errors. Figure 4C presents the correlation scatter plots between measured and predicted values of each performance index. The data points are closely distributed around the diagonal line with an extremely strong linear correlation, and the correlation coefficient R2 is close to 1, indicating a high agreement between predicted and measured values. This effectively improves the prediction validity and reliability of the models for specimens beyond the 17 groups of samples [36,37].

#### 3.1.3. Optimization and Validation

Response surface plots and contour plots were drawn to intuitively reveal the interactive effects among various influencing factors and their laws of influence on the performance response values of concrete, and the influence mechanism of each factor was clarified combined with regression model analysis. The contour plots and 3D surface plots of the quadratic model demonstrated the interactive results between factors [38].

##### Interactive Effects of Three Factors on Compressive Strength

**Figure 5:**
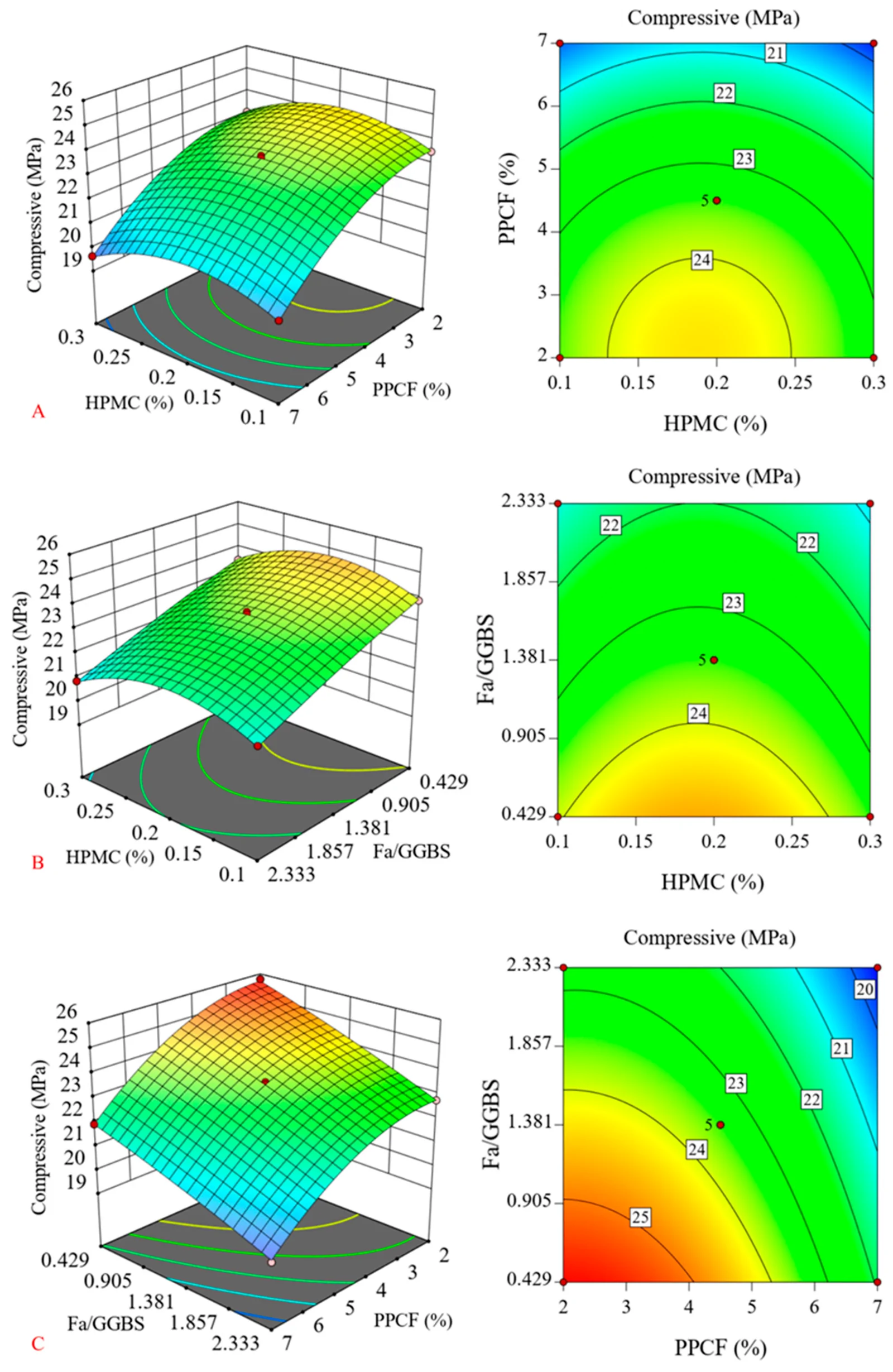
Response surface plots and contour plots of compressive strength for PPCF and HPMC (**A**), FA/GGBS and HPMC (**B**), and Fa/GGBS and PPCF (**C**).

As shown in Figure 5A, the response surface of the interaction between PPCF and HPMC presents a vaulted shape with an obvious extreme point. When the HPMC content is fixed, the compressive strength of concrete decreases significantly with the increase in PPCF content, which is mainly attributed to the fact that excessive fibers tend to form weak interfaces and introduce pores and microcracks, thereby reducing the compressive bearing capacity of the matrix. The effect of HPMC content on compressive strength is relatively gentle, with a peak appearing only at approximately 0.2% content, and the overall regulation effect is limited. As illustrated in Figure 5B, the response surface of the interaction between HPMC and FA/GGBS exhibits a gentle ridge shape. Under the condition of fixed HPMC content, compressive strength declines with the increase in the FA/GGBS blending ratio, which is closely related to the lower hydration activity of fly ash than that of GGBS and the insufficient hydration reaction of the system. The contour distribution is symmetric, and compressive strength first increases and then decreases with HPMC content, yet the overall fluctuation range is small, further verifying its weak regulation effect. As presented in Figure 5C, the response surface of the interaction between PPCF and FA/GGBS is a typical unimodal surface with a distinct extreme point. The increase in the content of both components leads to a gradual decrease in compressive strength, and the combination of low contents is more conducive to improving the compressive performance of concrete. According to the density of contour lines, the contours along the PPCF axis are denser with a steeper gradient, indicating that its negative impact on compressive strength is more significant, which stems from the sharp increase in interface defects caused by the high fiber content. The adverse effect of FA/GGBS is mainly due to the low hydration degree resulting from insufficient activity of fly ash.

##### Interactive Effects of Three Factors on Direct Shear Strength

**Figure 6:**
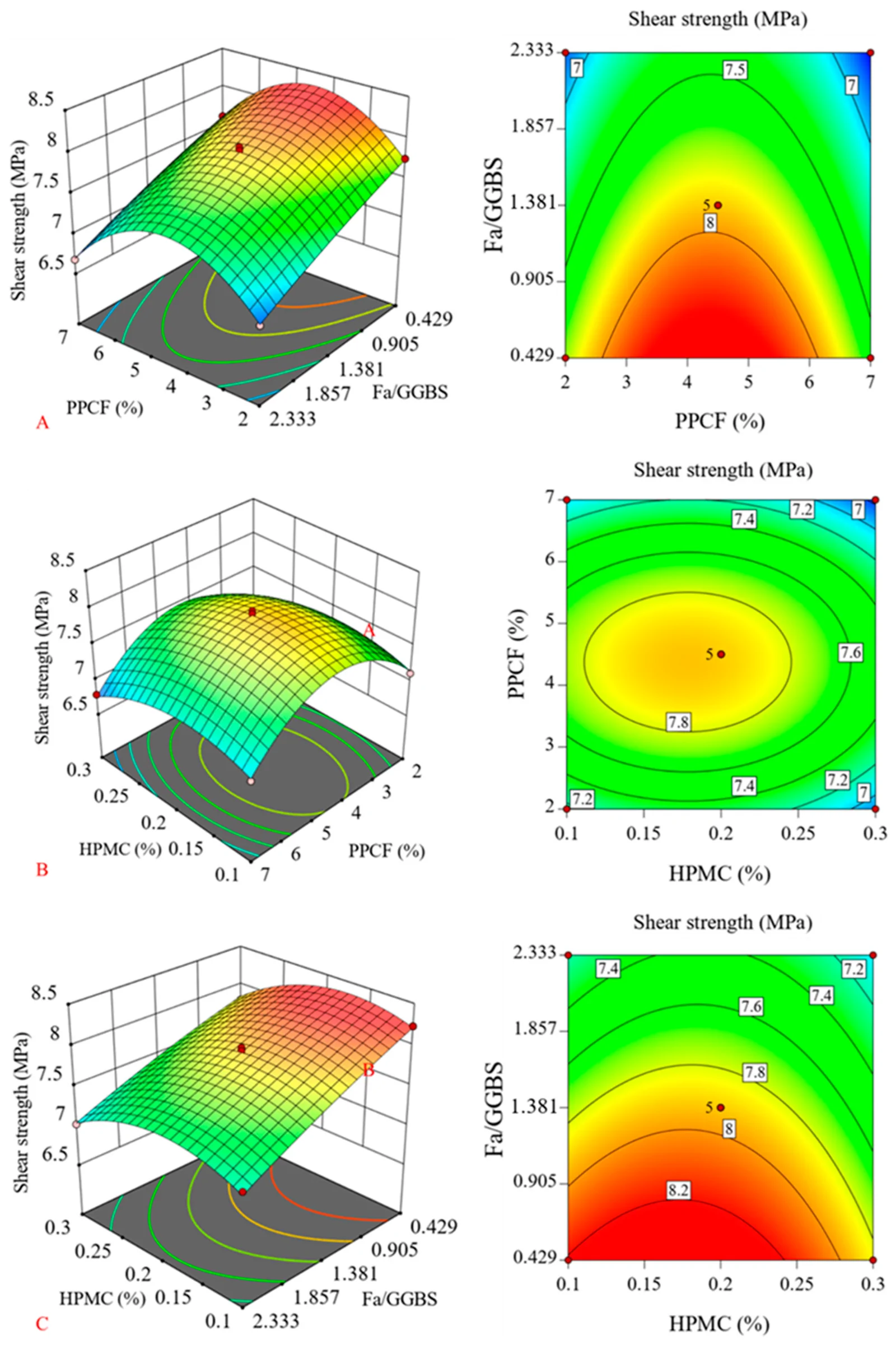
Response surface plots and contour plots of the direct shear strength for PPCF and HPMC (**A**), FA/GGBS and HPMC (**B**), and Fa/GGBS and PPCF (**C**).

Figure 6 shows the response surface and contour plots of the effects of PPCF, HPMC and FA/GGBS on the direct shear strength of concrete. As observed in Figure 6A, the response surface of the interaction between PPCF and FA/GGBS is distributed in a ridge shape. Along the PPCF content axis, the direct shear strength reaches the maximum peak in the middle and gradually decreases toward both sides. When the PPCF content is fixed, the direct shear strength of concrete shows a monotonically decreasing trend with the increase in the FA/GGBS blending ratio.

As displayed in Figure 6B, the response surface of the interaction between HPMC and PPCF presents a dome shape, where the direct shear strength peaks in the central region of the surface and gradually decreases toward the periphery. By comparing the contour distribution characteristics, the contours along the PPCF content axis are denser with a steeper gradient, while those along the HPMC content axis are relatively sparse and gentle, indicating that the influence degree of PPCF on the direct shear strength of concrete is significantly higher than that of HPMC. This result is consistent with the toughening and strengthening mechanism of fibers in concrete: PPCF can restrain the initiation and propagation of cracks in the matrix through bridging effect, directly improving the shear-bearing capacity. HPMC, mostly used as a processing admixture, improves the workability of the paste and has limited direct contribution to mechanical strength. As shown in Figure 6C, the response surface of the interaction between HPMC and FA/GGBS takes the form of a gentle ridge, and their influence laws on direct shear strength differ considerably. An obvious extreme value of direct shear strength appears when the HPMC content ranges from 0.15% to 0.25%, suggesting that an appropriate amount of HPMC can improve the homogeneity of the paste, indirectly optimize the matrix structure and enhance the shear strength, whereas excessive incorporation exerts adverse effects. From the perspective of contour density, the contours along the HPMC axis are sparse with a gentle gradient, demonstrating that its influence amplitude on direct shear strength is slight and it is not the key factor controlling the shear performance of concrete.

##### Interactive Effects of Various Factors on Chloride Ion Electric Flux

**Figure 7:**
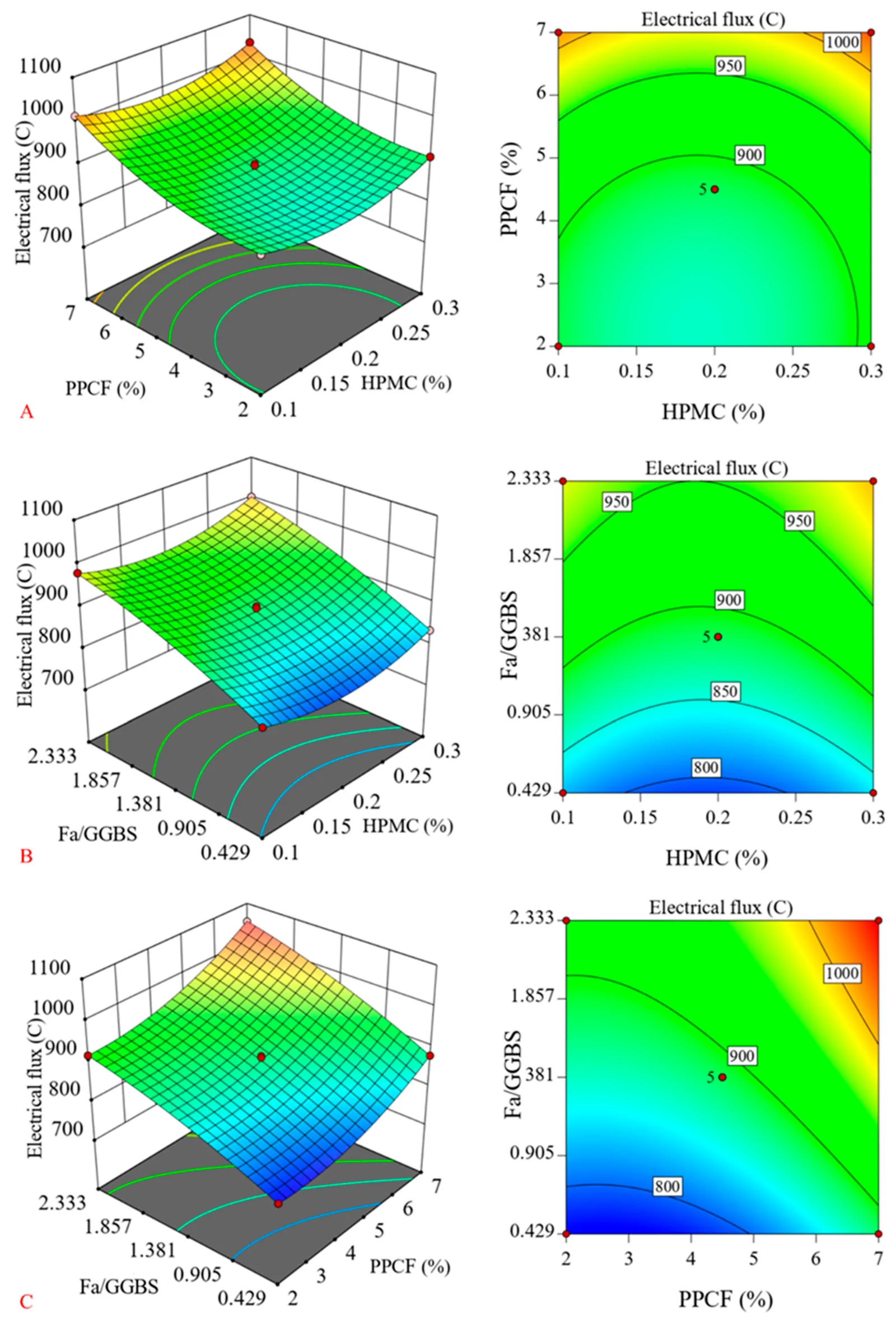
Response surface plots and contour plots of the anti-chloride ion permeability responses of PPCF and HPMC (**A**), FA/GGBS and HPMC (**B**), and FA/GGBS and PPCF (**C**).

Figure 7 depicts the response surface and contour plots of the effects of PPCF, HPMC and FA/GGBS on the chloride ion electric flux in concrete. As seen in Figure 7A, the response surface of the interaction between HPMC and PPCF is low in the middle and high on both sides. The chloride ion electric flux first decreases and then increases with the rise in HPMC content, and the contours are approximately semi-elliptical, indicating that a proper dosage of HPMC can optimize the impermeability of concrete, while over-dosage generates negative effects. Meanwhile, the chloride ion electric flux rises gradually with the increase in PPCF content, and PPCF exerts a more significant influence on electric flux.

Figure 7B presents the response surface and contours of the interaction between FA/GGBS ratio and HPMC. The surface shows a valley shape with high ends and a gentle middle part, and the surface along the FA/GGBS ratio axis has a larger inclination amplitude, meaning that this ratio has a remarkable effect on chloride ion electric flux. The lower the FA/GGBS ratio, the smaller the chloride ion electric flux in specimens and the stronger the chloride penetration resistance of concrete. Figure 7C reflects the influence laws of FA/GGBS and PPCF on chloride ion electric flux, with similar variation trends: the electric flux shows an upward tendency with the increase in content or ratio. Combined with the contour gradient variation, the influence degree of the FA/GGBS ratio on electric flux is higher than that of PPCF. A lower chloride ion electric flux represents greater resistance to chloride ion migration and superior impermeability and durability of concrete. Therefore, the adoption of low PPCF content and a low FA/GGBS ratio can effectively reduce chloride ion electric flux and enhance the chloride-penetration resistance of concrete. The mechanism lies in the fact that a low FA/GGBS ratio corresponds to a higher proportion of high-activity GGBS in the system, enabling the full play of the pozzolanic effect; meanwhile, low PPCF content can reduce interface defects and cut down harmful permeable channels.

#### 3.1.4. Optimal Mix Proportion Optimization and Validation

**Figure 8:**
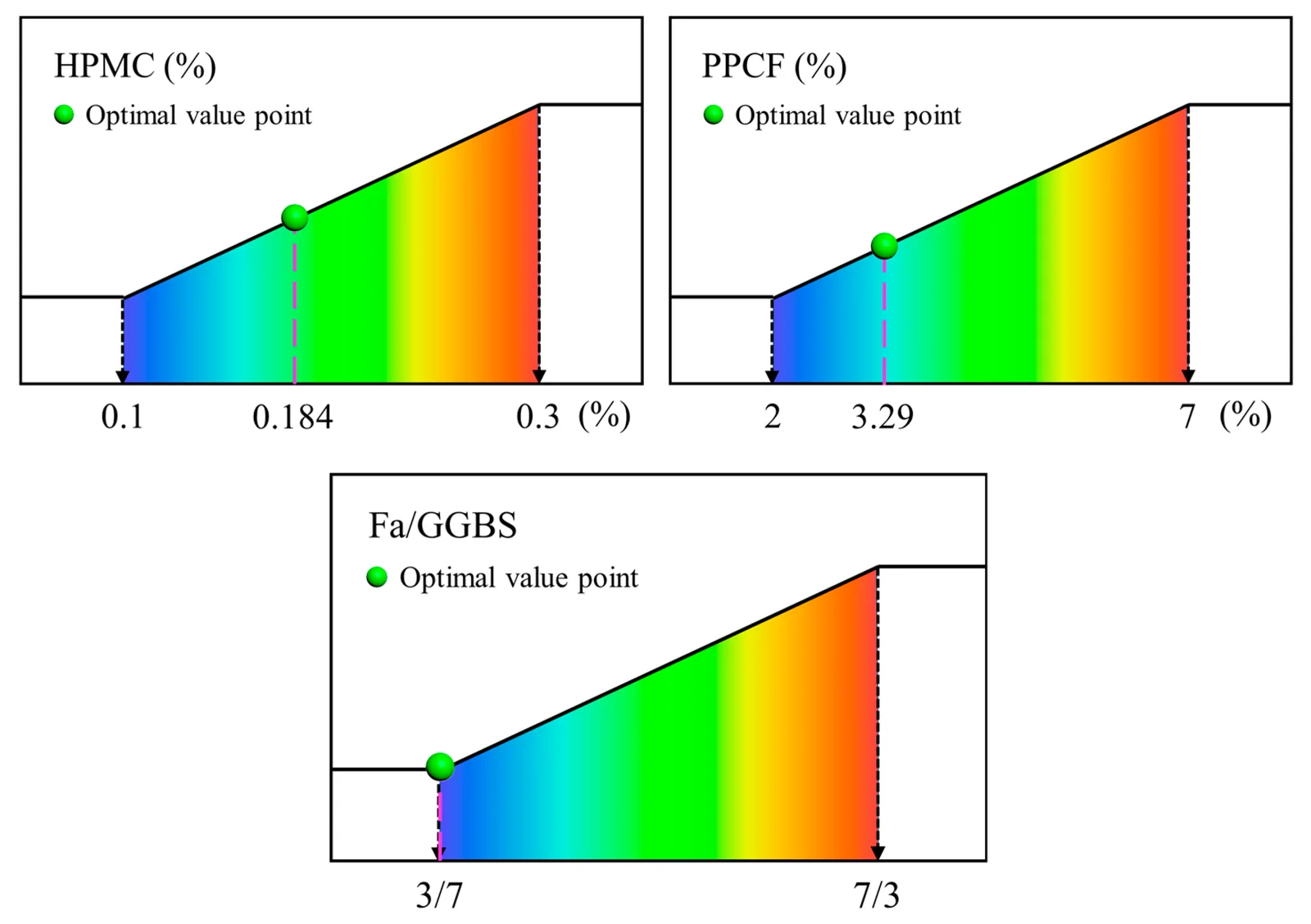
Optimized solution.

**Figure 9:**
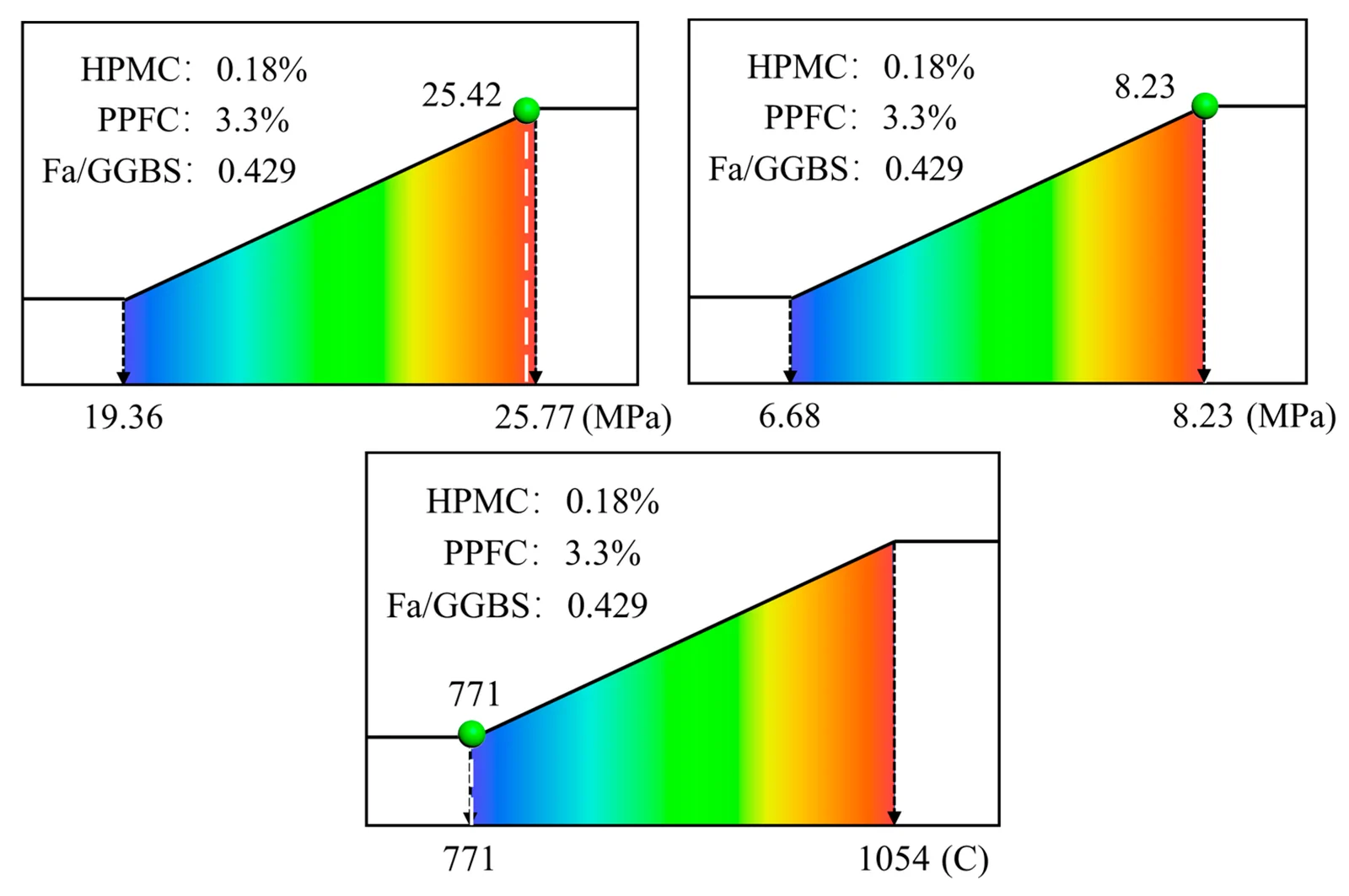
Predicted values of the optimization scheme.

To overcome the limitation that response surface and contour plots only allow qualitative analysis, the Design-Expert software was employed to quantitatively solve the regression models, so as to accurately determine the optimal process parameters [39]. The optimization objectives were set as follows: to increase compressive strength (25.42 MPa) and direct shear strength (8.23 MPa) while reducing chloride ion electric flux (771 C), thereby achieving the synchronous optimization of load-bearing capacity and impermeability durability of the material. During the optimization, the variable ranges were set as HPMC (0.1–0.3%), PPCF (3–7%) and FA/GGBS (3/7–7/3) (Figure 8). Through multi-objective optimization calculation by the software, the final optimal mixing ratio combination was obtained as follows: HPMC = 0.18%; PPCF = 3.3%; FA/GGBS = 3/7 (0.429) (Figure 9).

To verify the reliability of the optimal process, validation experiments were carried out: material specimens were prepared according to the optimal mixing ratio, and their actual compressive strength, direct shear strength and electric flux were tested. The percentage error formula (Equation (6)) was used to evaluate the error between predicted and experimental results. If all error values were less than 5%, it indicated that the optimal process parameters were in good agreement with the actual performance.

(6) $$\delta =\left|\frac{\vartheta_{E}-\vartheta_{P}}{\vartheta_{P}}\right|\times 100\%$$

where δ = percentage error (%); $\vartheta_{E}$ = experimental measured value; and $\vartheta_{P}$ = model predicted value.

The validation experimental results are listed in Table 8. It can be seen that the errors between measured and predicted values for compressive strength, direct shear strength and chloride ion electric flux are 3.36%, 2.63% and 4.02%, respectively, all of which are less than 5%. This demonstrates that the optimized mix proportion parameters are reliable and accurate, and can meet the performance requirements of shotcrete for deep gold mine roadways.

### 3.2. Experimental Comparative Analysis

#### 3.2.1. Compressive Performance

**Figure 10:**
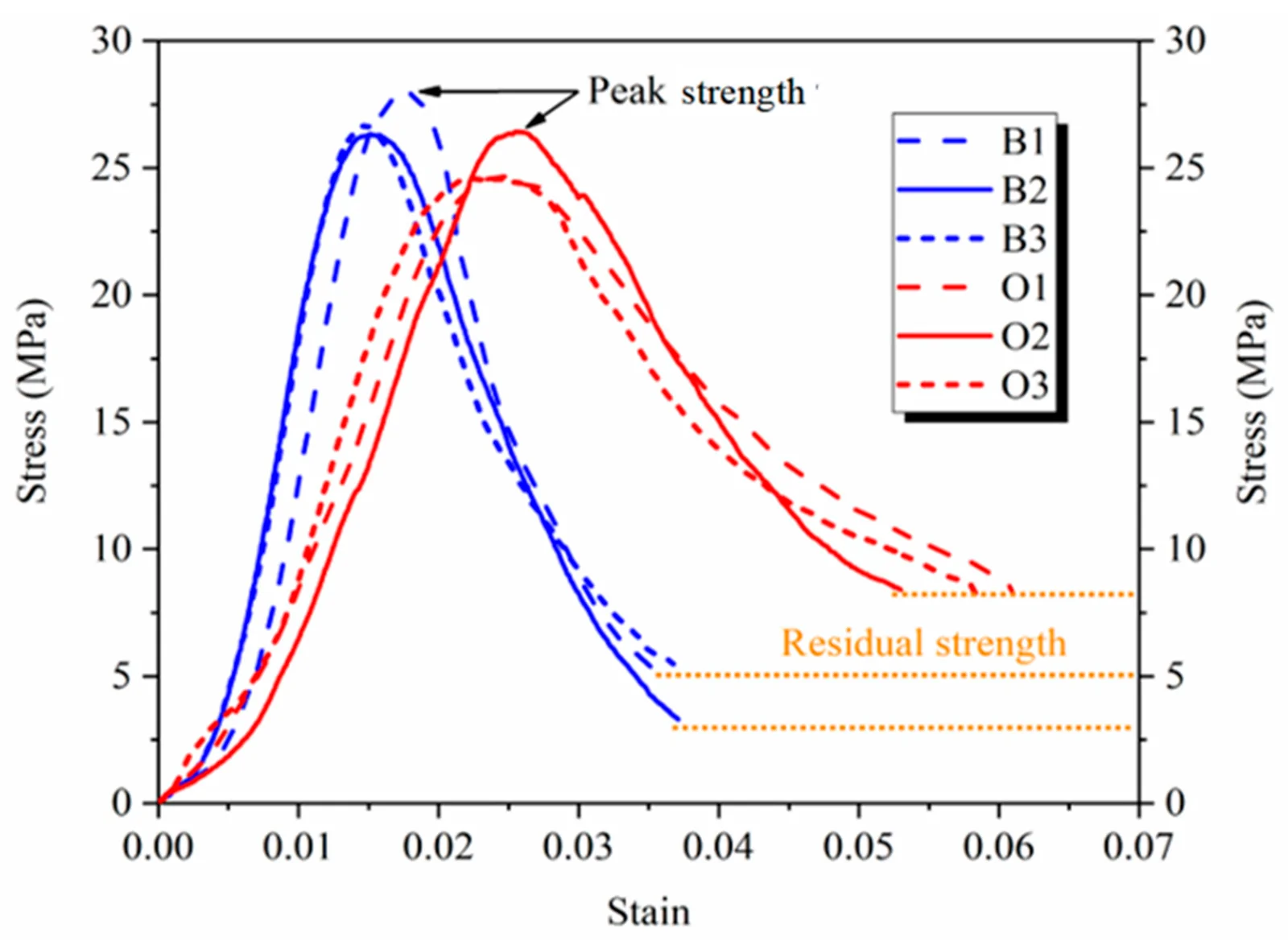
Compressive strength of the two material groups.

**Figure 11:**
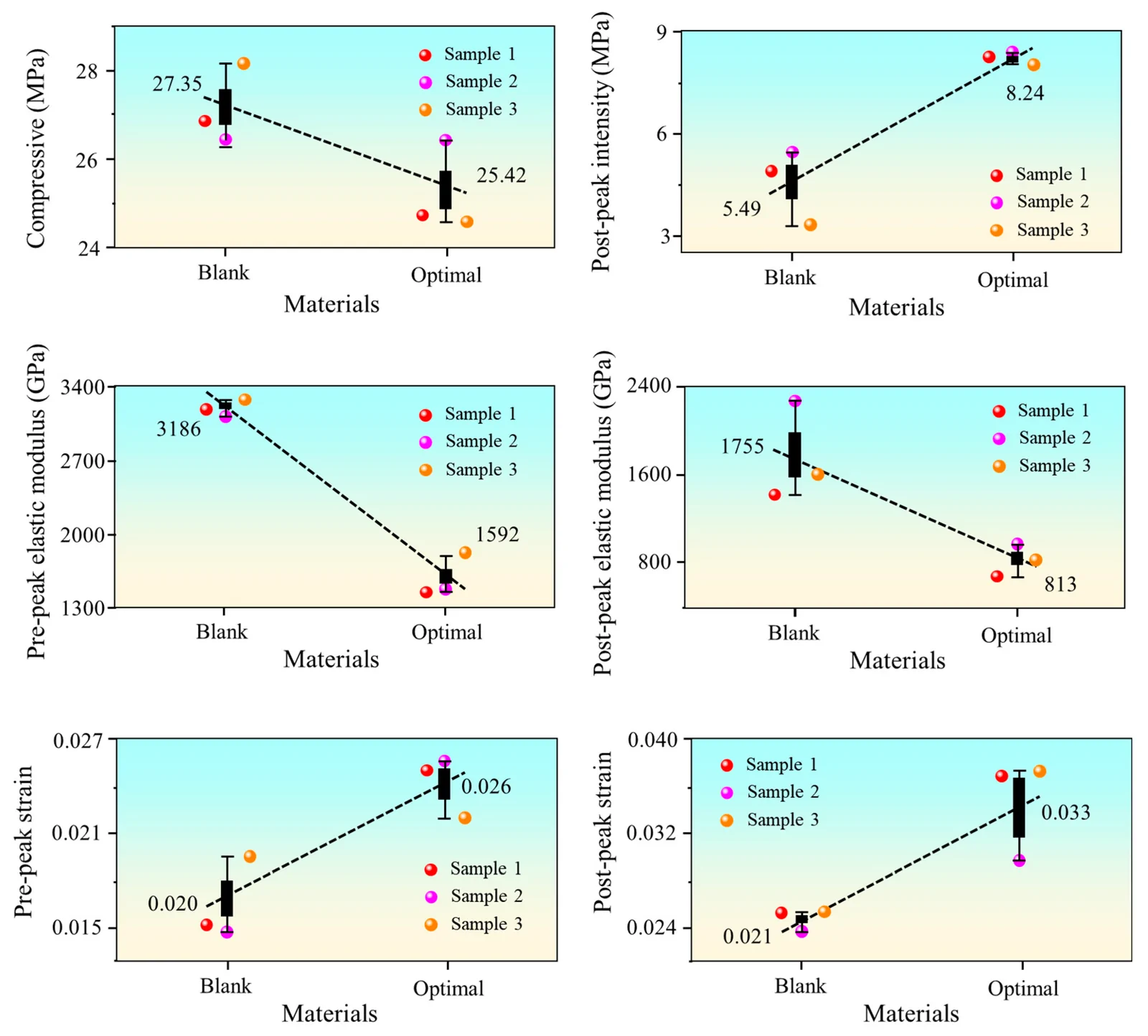
Scatter plots of mechanical properties of the two material groups (The dotted line in the figure connects the mean values of the two groups of data and is used to visually indicate the direction and magnitude of change between the two groups).

Compressive strength tests can accurately characterize the peak load-bearing capacity, strain evolution, and residual strength after failure of concrete under uniaxial compression, providing critical experimental data for elucidating the resistance of support materials to crushing failure and optimizing mix designs [40]. As shown in Figure 10 and Figure 11, the compressive properties of ordinary concrete prepared with mine water (Blank group) were compared with those of concrete with an optimized mix design. The Blank group exhibited a peak compressive strength of 28.04 MPa and a residual strength of 4.85 MPa, with pre-peak and post-peak strains of 0.0019 and 0.0025, respectively, corresponding to the pre-peak and post-peak elastic moduli of 27.97 GPa and 14.46 GPa. In comparison, the peak compressive strength of the Optimal group decreased to 25.24 MPa, a reduction of 9.98%; this change resulted from the substitution of mineral admixtures affecting the hydration rate. At the same time, the residual strength of the Optimal group increased significantly to 8.24 MPa, representing a 41.14% increase over the baseline group. This was primarily due to the three-dimensional bridging effect of polypropylene coarse fibers: during compressive deformation, the fibers dissipate energy through pull-out and fracture, thereby inhibiting the rapid propagation of microcracks and delaying post-peak stress decay, and thus maintaining a higher residual load-bearing capacity. In terms of deformation characteristics, the pre-peak and post-peak strains in the Optimal group increased to 0.0024 and 0.0033, respectively, representing increases of 26.3% and 32%, reflecting a significant improvement in the ductility of the optimized system. Furthermore, the pre-peak and post-peak elastic moduli decreased to 16.70 GPa and 7.20 GPa, representing reductions of 40.29% and 50.21%, respectively. This is because the film-forming effect of HPMC enhances the bond strength between the fibers and the matrix, resulting in more uniform stress transfer at the interface. At the same time, the introduction of fibers increases the matrix’s capacity for plastic deformation. Together, these factors reduce the material’s stiffness, making it better suited to accommodate the creep deformation of the surrounding rock. In terms of the stress–strain curve profiles, the Blank group (blue curve) exhibited typical brittle fracture characteristics, with a sharp drop in stress after reaching the peak; in contrast, the Optimal group (red curve) showed a more gradual decay in stress after the peak and higher residual strength, further confirming the toughening effect of multi-component synergistic modification [41].

**Figure 12:**
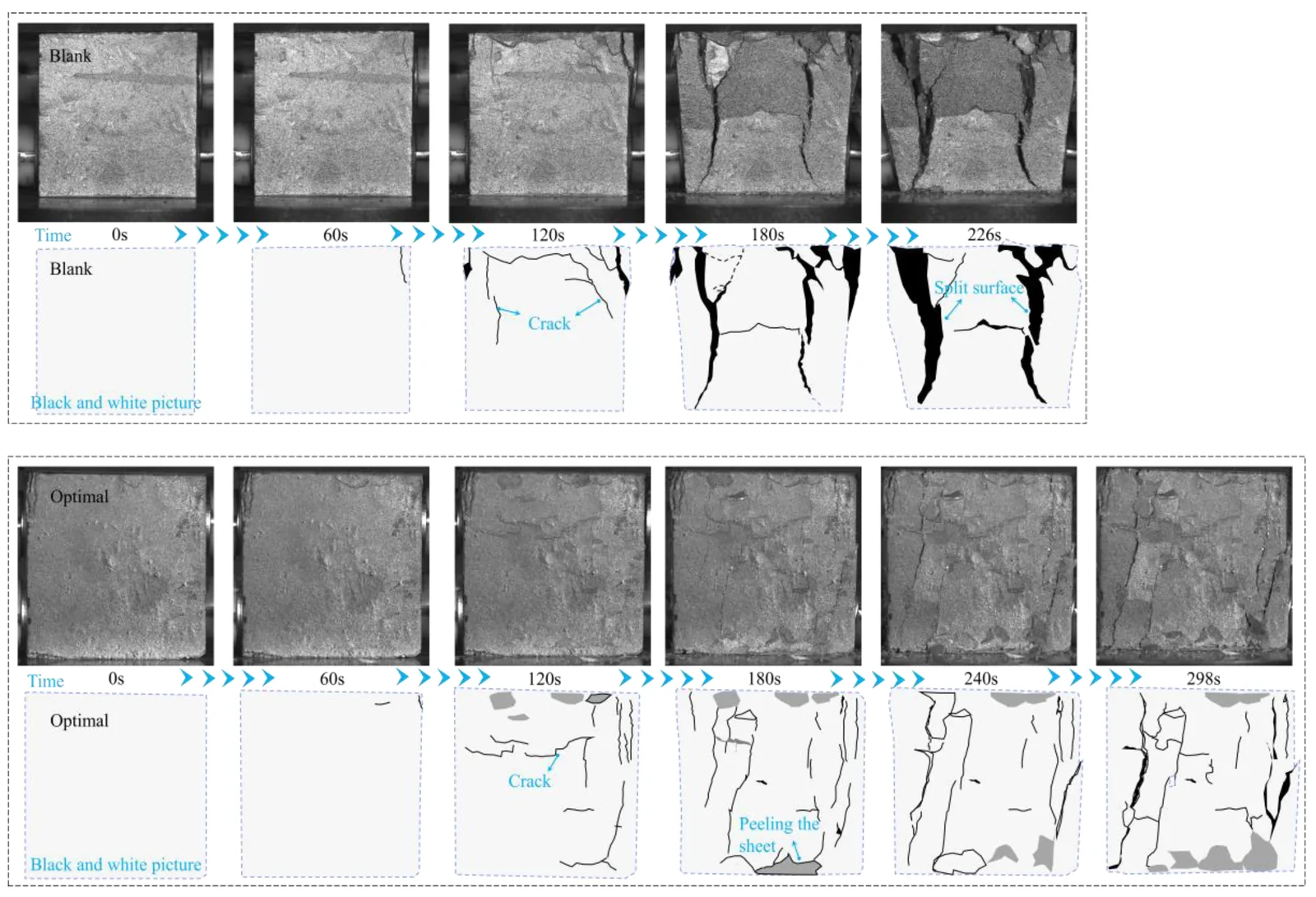
Compressive failure processes of the two material groups.

The failure characteristics of the two groups of specimens were further plotted (Figure 12). Under uniaxial compression, the plain concrete specimens prepared with mine water presented typical brittle splitting failure: no obvious macroscopic cracks appeared on the specimen surface at the initial loading stage (0–2 min); with the increase in load, 2–3 main cracks generated along the axial direction at 3–4 min and propagated rapidly. At final failure, the main cracks penetrated the entire specimen, accompanied by the spalling of a small amount of debris. The failure mechanism was that internal microcracks in the matrix rapidly propagated and penetrated along the axial direction under stress concentration, forming main splitting surfaces, and showing obvious brittle fracture characteristics.

In contrast, the optimized concrete exhibited ductile spalling–cracking coupled failure: no obvious cracks were observed on the surface at the initial loading stage (0–2 min); flaky spalling zones (gray areas) first appeared at 3–4 min, followed by the initiation of microcracks at the edges of the spalling zones; microcracks gradually propagated and interweaved into a network at 5–6 min. No penetrating main splitting surface formed at final failure, and the failure was dominated by the coordinated development of multi-regional flaky spalling and network cracks. The failure mechanism originated from the synergistic effect of composite cementitious materials, fibers and a water-retaining agent: the bridging effect of PPCF retarded crack propagation, and the water-retention effect of HPMC improved interfacial bonding strength, enabling the specimen to release energy through flaky spalling under loading and avoiding brittle splitting, thus presenting superior ductile failure characteristics.

#### 3.2.2. Shear Performance

**Figure 13:**
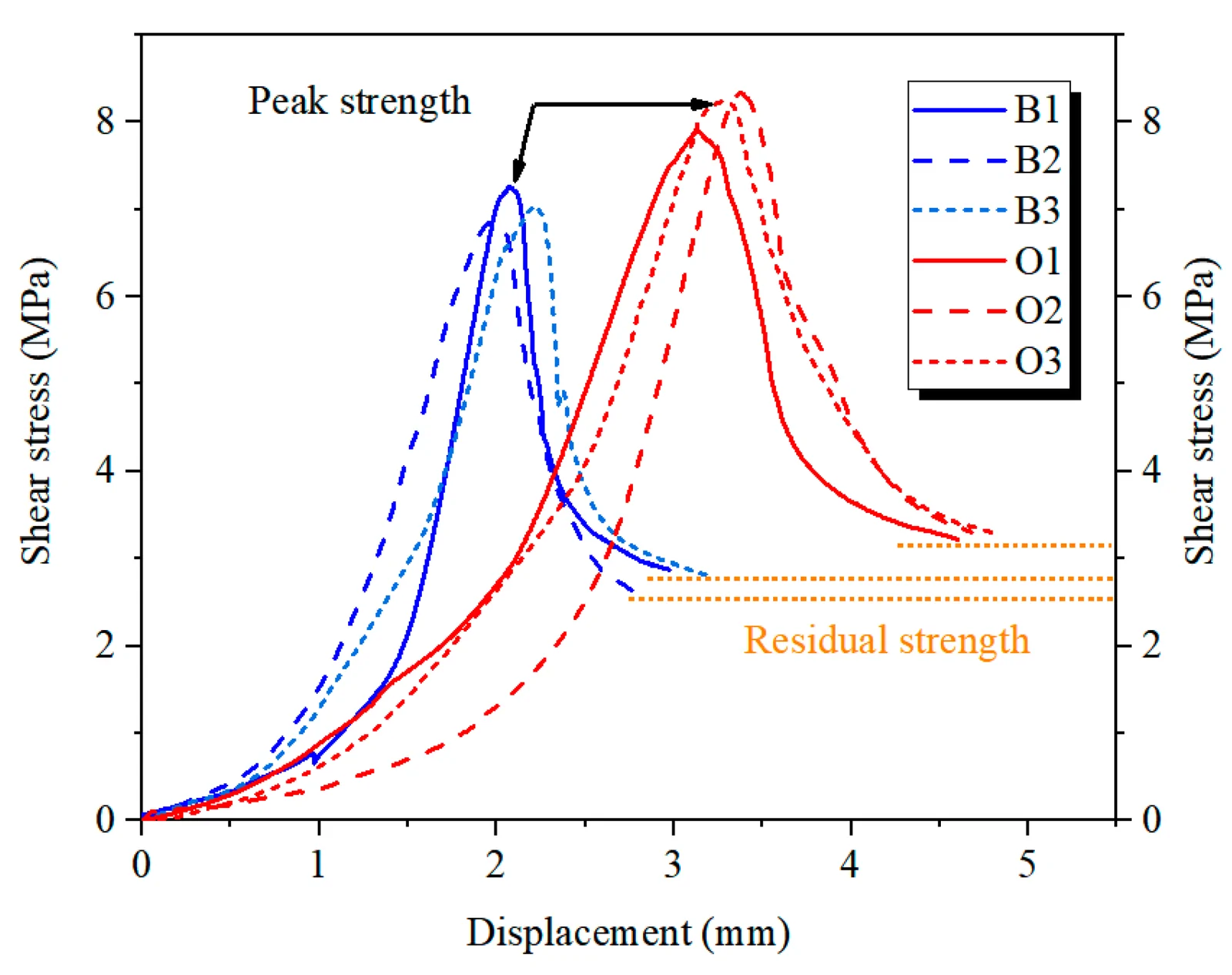
Direct shear test results of the two material groups.

**Figure 14:**
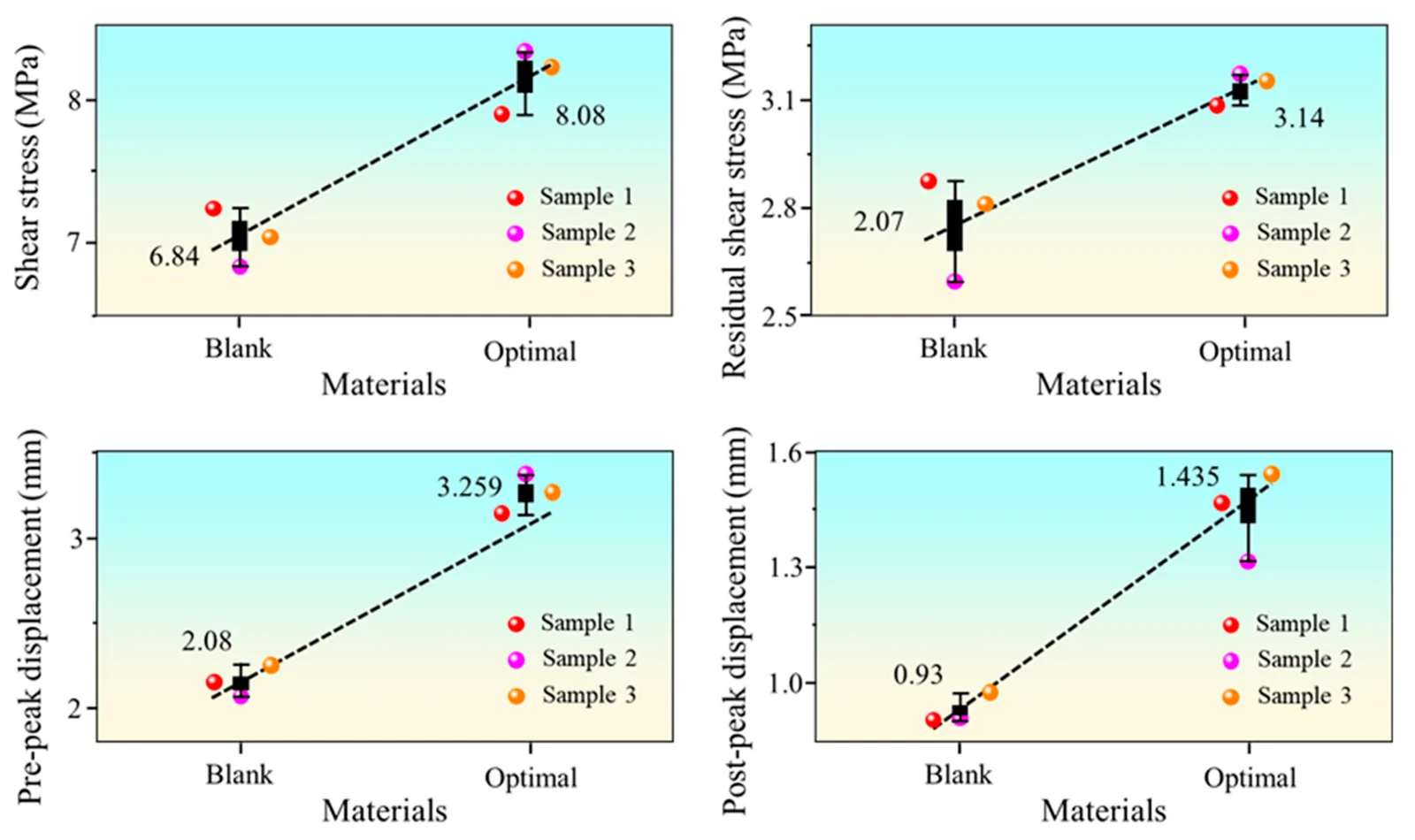
Mechanical properties of the two material groups in direct shear tests (The dotted line in the figure connects the mean values of the two groups of data and is used to visually indicate the direction and magnitude of change between the two groups).

Direct shear tests serve as a core method to quantify the shear strength, deformation characteristics and residual bearing capacity of concrete [42,43]. Combined with the experimental results shown in Figure 13 (shear stress–displacement curves) and Figure 14 (scatter plots of shear performance), a quantitative comparison was conducted between the ordinary concrete prepared with mine water (Blank group) and the optimized concrete (Optimal group): The Blank group had a peak shear stress of only 6.84 MPa and a residual shear strength of 2.07 MPa, with pre-peak and post-peak displacements of 0.906 mm and 0.93 mm respectively. The peak shear stress of the Optimal group was significantly increased to 8.33 MPa (an increase of 21.8%), and the residual shear strength rose to 3.14 MPa (an increase of 51.7%). The pre-peak and post-peak displacements increased to 3.259 mm and 1.435 mm respectively. From the curve morphology, the Blank group (blue curve) showed rapid stress attenuation after the peak, representing brittle failure; the Optimal group (red curve) had a milder post-peak stress decay and higher residual strength, indicating that the optimized system possessed better ductility and resistance to shear deformation.

**Figure 15:**
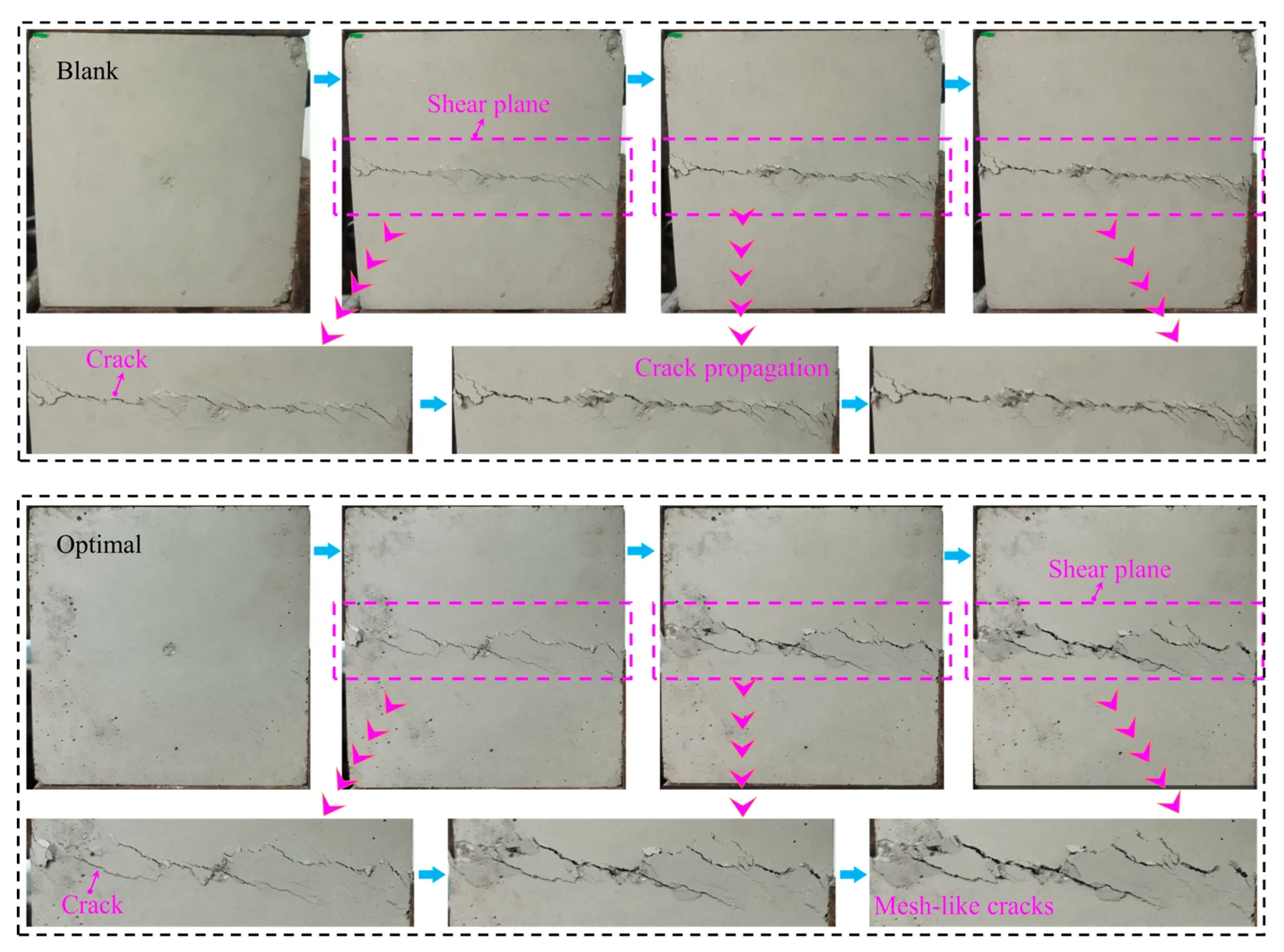
Direct shear failure processes of the two material groups.

The failure characteristics of direct shear tests are presented in Figure 15. The Blank group displayed typical brittle shear failure: a single continuous main crack initiated rapidly at the initial loading stage, then quickly penetrated the shear plane, and eventually, obvious slippage occurred along the crack. The failure was concentrated and irreversible, reflecting the brittle nature of easy rapid crack propagation without energy dissipation. In the Optimal group, multiple dispersed microcracks initiated at the initial loading stage, and subsequent cracks interweaved without forming a penetrating main crack. The final failure mode was a composite morphology of network cracks and local spalling. The difference in failure modes between the two groups was attributed to multi-component synergistic modification: the randomly distributed polypropylene coarse fibers formed a three-dimensional constraint network, transferred stress via the bridging effect, inhibited microcrack propagation, delayed post-peak stress attenuation, and significantly improved residual strength and deformability under shear deformation.

#### 3.2.3. Impermeability Performance

**Figure 16:**
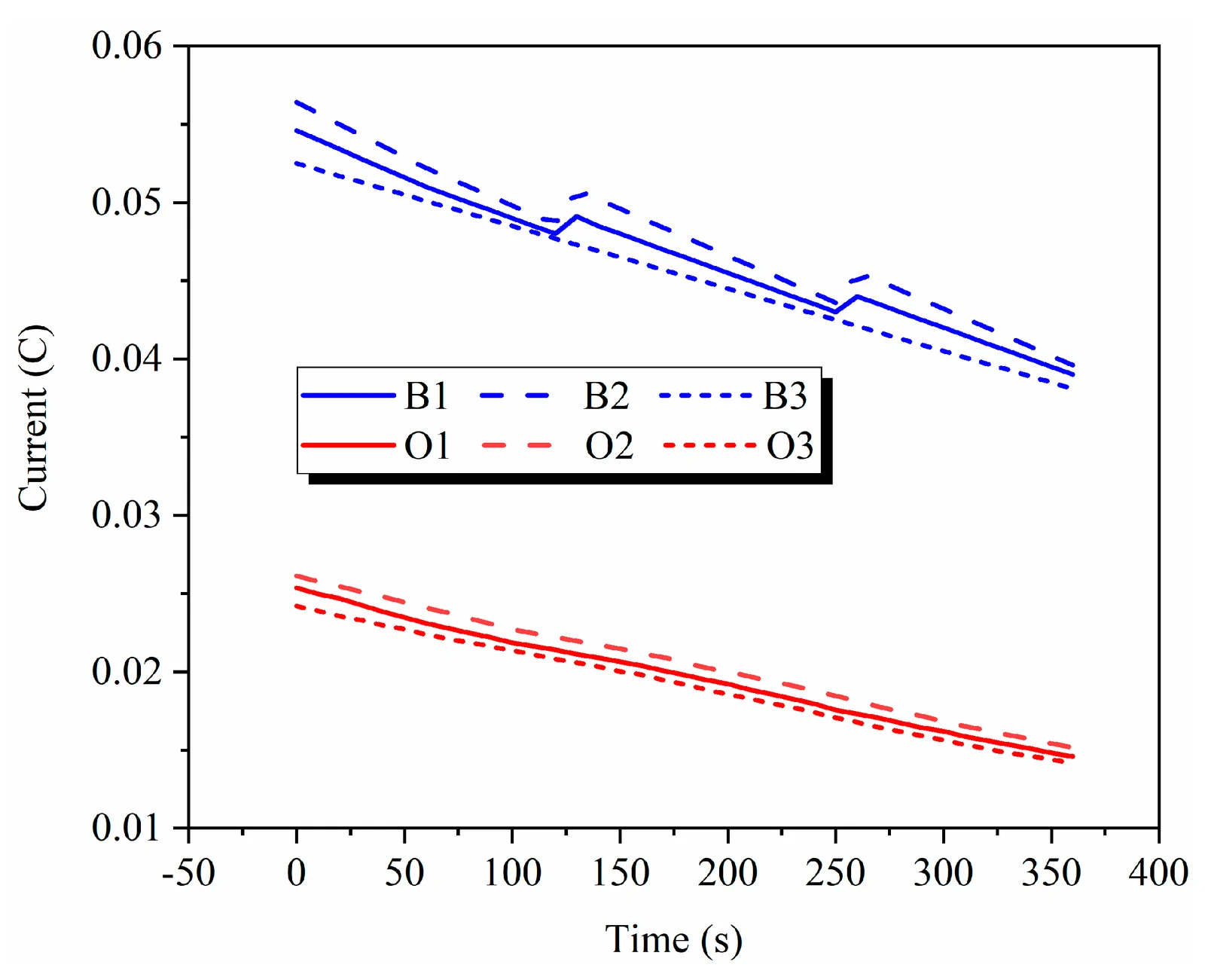
Relationship between time and current for the two concrete groups.

**Figure 17:**
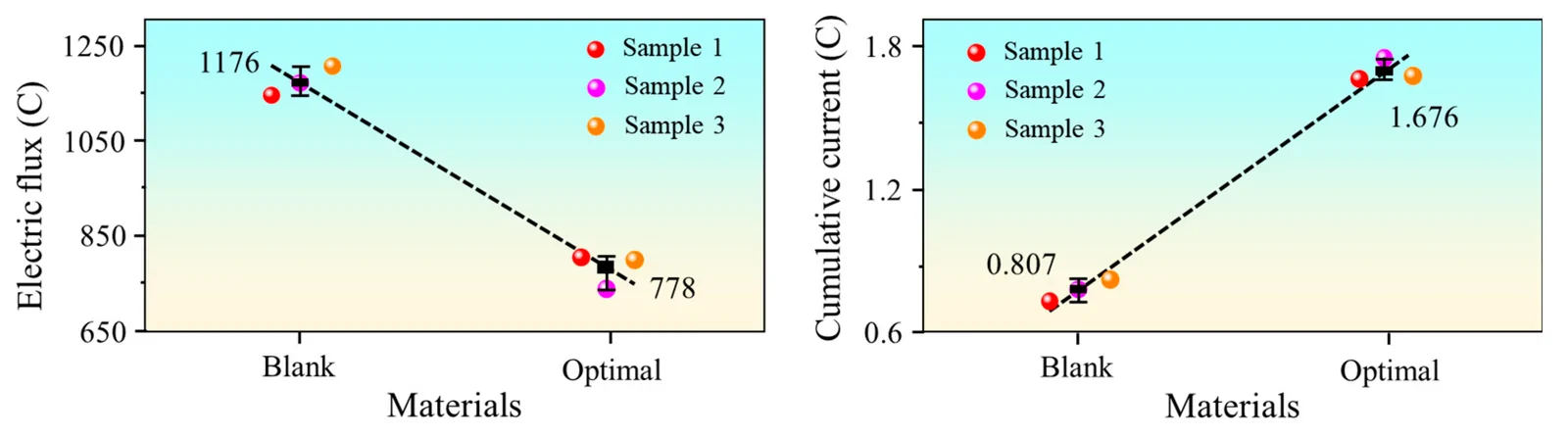
Relationship between electric flux and cumulative current for the two concrete groups (The dotted line in the figure connects the mean values of the two groups of data and is used to visually indicate the direction and magnitude of change between the two groups).

The chloride ion electric flux test, as a core method to quantify the ion permeability of concrete, can rapidly characterize the durability of support materials in the underground corrosive environment [44]. The experimental data (Figure 16 and Figure 17) show the following. In the 6 h direct current electro-osmosis test, the initial current of the Blank group concrete was 0.025–0.057 A, while that of the Optimal group increased to 0.052–0.057 A, and both groups exhibited a stable attenuation trend over time. Although the Optimal group had a higher initial current, its current decay rate was noticeably slower than that of the Blank group, and the cumulative current increased from 0.807 C (Blank group) to 1.676 C. More importantly, the electric flux test results revealed that the 6 h electric flux in the Blank group concrete was 1176 C, while that in the Optimal group decreased significantly to 778 C, representing a charge reduction of approximately 33.8%, which indicates an improved resistance to chloride ion transport. The microscopic mechanism is as follows: the pozzolanic reaction of fly ash and GGBS can immobilize chloride ions through chemical binding. Reactive Al_2_O_3_ in GGBS combines with chloride ions to form highly stable Friedel’s salt, and the C-S-H gel generated by fly ash reduces the concentration of free chloride ions via physical adsorption. These dual mechanisms effectively block chloride diffusion pathways. Meanwhile, the film-forming effect of HPMC strengthens the bonding between fibers and the cementitious matrix, significantly reducing permeable paths from interfacial defects and further improving the impermeability of the material.

## 4. Engineering Experiment

**Figure 18:**
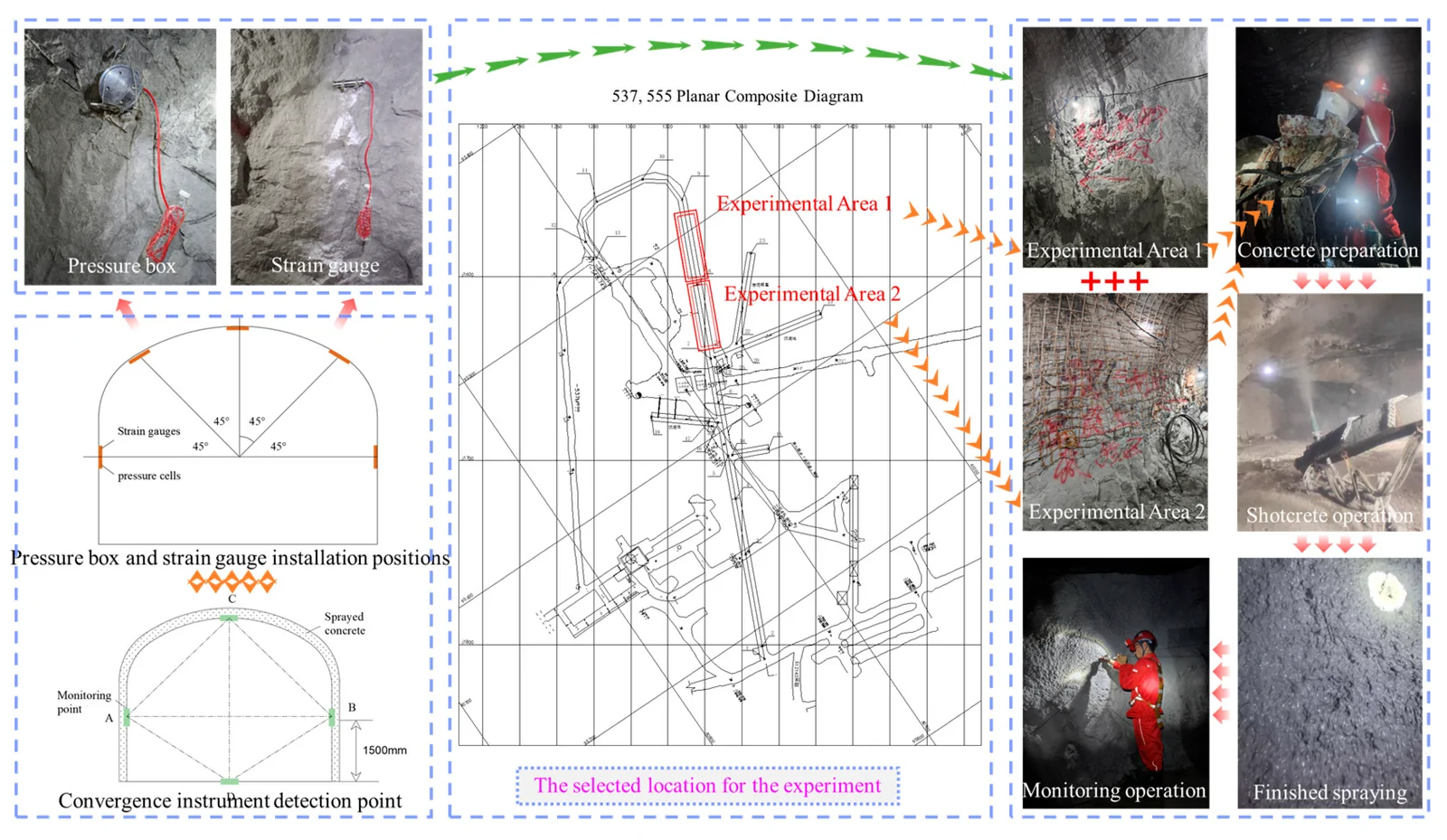
Layout of field test design, equipment installation and shotcrete support.

**Figure 19:**
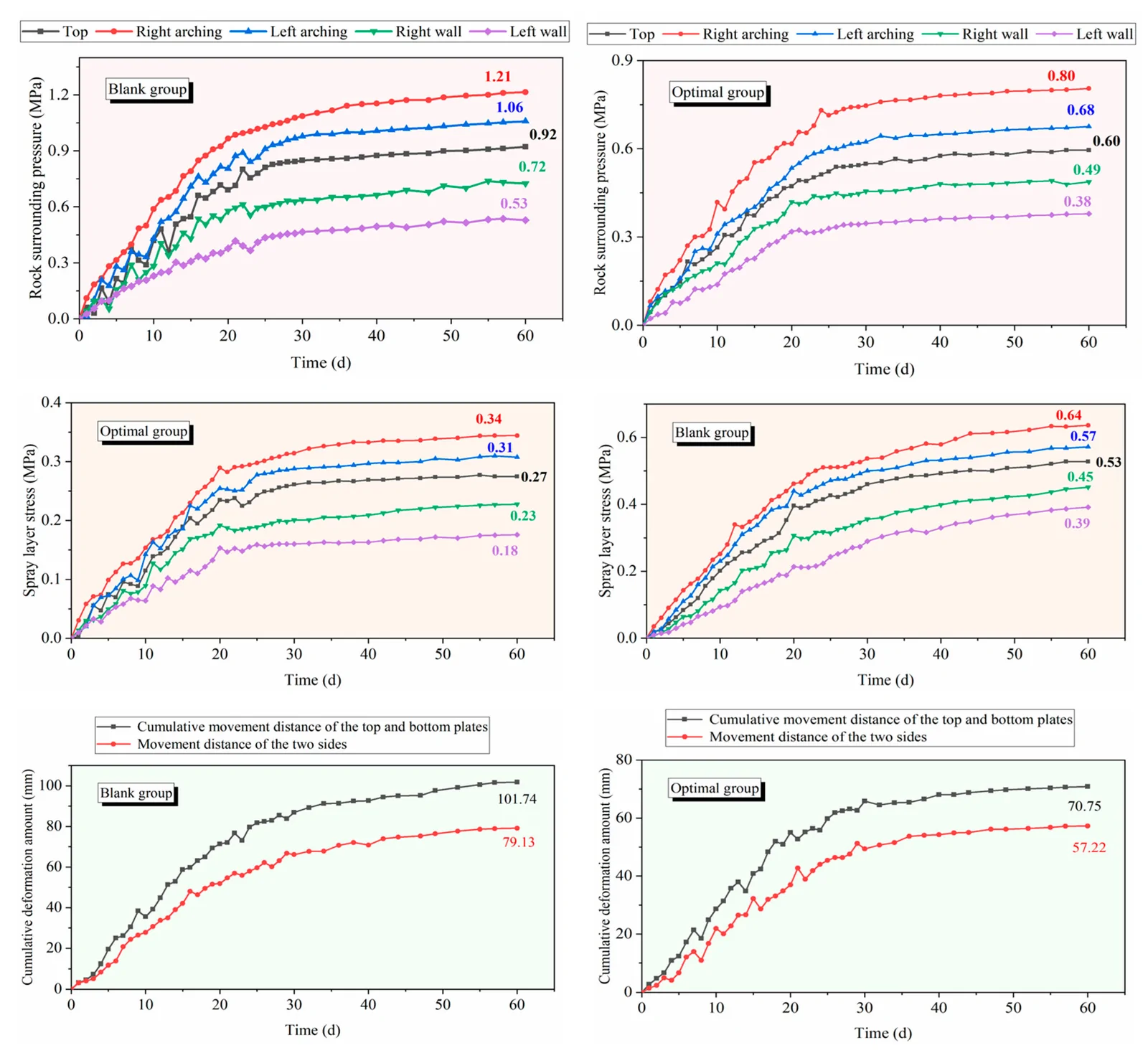
Comparison of surrounding rock pressure, shotcrete stress and roadway convergence between the two shotcrete types in field tests.

To verify the engineering adaptability, construction feasibility and long-term service performance of the optimized shotcrete, on-site comparative tests were carried out at a depth of 537 m, relying on the deep roadway project of the Sanshandao Gold Mine. Two roadways with consistent geological conditions and a length of 40 m each were selected as test sections: Test Section 1 was supported by ordinary shotcrete, and Test Section 2 by the optimized Optimal group shotcrete. A multi-parameter continuous monitoring system was established using PCE0270 vibrating wire pressure cells, RCE0350 embedded strain gauges and SLJ-1 digital convergence meters. Surrounding rock pressure, shotcrete layer stress and roadway surface displacement were continuously monitored for 2 months (Figure 18), with the monitoring results shown in Figure 19.

The monitoring results in Figure 19 indicate that for Test Section 1 supported by ordinary concrete, the peak surrounding rock pressure at the vault was 1.21 MPa, the peak shotcrete layer stress reached 0.64 MPa, the cumulative convergence deformation of the two sidewalls was 79.13 mm, and the cumulative deformation of the roof and floor was 107.74 mm. This support system presented typical rigid bearing characteristics with weak deformation coordination ability, which made it difficult to adapt to the creep deformation of surrounding rock. Obvious water seepage and dripping occurred on the roadway wall, eventually leading to local cracking of the support structure. For Test Section 2 supported by the optimized concrete, the surrounding rock pressure at each measuring point decreased by about 33.9% compared with Test Section 1, with the peak vault surrounding rock pressure being only 0.80 MPa and the peak shotcrete layer stress reduced to 0.34 MPa. The cumulative sidewall convergence was 57.22 mm and the cumulative roof-floor deformation was 70.75 mm, representing decreases of 27.7% and 34.3% respectively, demonstrating excellent ductile yielding and pressure-relieving characteristics. No obvious water seepage or large wet areas were observed on the roadway wall of Test Section 2. Comprehensive analysis shows that ordinary shotcrete has high rigidity and poor deformation adaptability, which cannot accommodate the continuous creep of surrounding rock in deep roadways. Meanwhile, its weak impermeability allows mine water and chloride ions to easily intrude along internal defects, accelerating structural degradation. On the one hand, the optimized shotcrete relies on the ductile toughening and energy-dissipating yielding effects of polypropylene fibers to effectively dissipate the deformation energy of surrounding rock, alleviate local stress concentration, and improve the coordinated deformation capacity between the support structure and surrounding rock. On the other hand, the fly ash–GGBS composite cementitious system optimizes the composition of cement hydration products and refines the microporous structure of the matrix, effectively blocking the intrusion pathways of mine water and chloride ions. The synergistic improvement in mechanical bearing capacity, impermeability and corrosion durability is thus achieved.

## 5. Influence Mechanism of Modified Components on Mechanical and Impermeability Properties of Concrete

High-salinity mine water in coastal gold deposits intrudes into the concrete matrix through capillary penetration, damaging hydration products and causing surface spalling, and accelerating the initiation and penetration of microcracks. This eventually leads to strength degradation of the shotcrete body and cracking of the support structure, threatening underground safety. Traditional shotcrete, with its loose hydration structure and high porosity, lacks corrosion resistance and crack resistance, making it unsuitable for high-salinity extreme conditions.

FA and GGBS are the core components for cementitious material modification. Both possess pozzolanic activity and micro-aggregate effects. The single incorporation of FA retards cement hydration and reduces strength, but composite blending with high-activity GGBS can compensate for this defect. An appropriate amount of FA-GGBS undergoes pozzolanic reactions with Ca(OH)_2_, a cement hydration product, generating dense low-Ca/Si-ratio C-S-H gel. This not only strengthens the aggregate–matrix interface, reduces pores and oriented Ca(OH)_2_ crystals, and improves interfacial bonding strength, but also refines pores to optimize stress transfer. Although the slightly slower early hydration results in a small decrease in peak compressive strength, the long-term strength stability and ductility are significantly enhanced [45,46,47,48]. Meanwhile, reactive Al_2_O_3_ in GGBS combines with Cl^−^ to form stable Friedel’s salt, and the C-S-H gel from FA adsorbs free chloride ions via its high specific surface area. These dual effects block the transport of Cl^−^ and SO_4_^2−^, greatly reducing electric flux and strengthening corrosion resistance.

In addition, randomly dispersed polypropylene coarse fibers (PPCF) can bridge microcracks initiated on the shear plane, transfer shear stress through the bridging effect, and hinder crack propagation and penetration. Under shear loading, fibers dissipate energy through pull-out and fracture, transforming the failure mode from “direct penetration of the shear plane” in the Blank group to the ductile failure, thereby improving the material’s ability to adapt to surrounding rock creep deformation. However, the PPCF content must be reasonably controlled: excessive fibers introduce additional interfacial pores into the matrix, impair compactness, and tend to form local stress concentration zones, leading to a decline in peak compressive strength. Insufficient interfacial bonding between fibers and the matrix allows high-salinity mine water to easily penetrate along the fiber-matrix interface, forming preferential corrosion channels and conversely weakening the corrosion resistance of the material.

HPMC ensures the modification effect through interfacial strengthening and hydration regulation: its viscosity-increasing and water-retention properties improve the workability of shotcrete and guarantee uniform dispersion of PPCF and FA-GGBS. The film-forming effect creates a dense hydration film at the fiber-matrix and aggregate interfaces, enhancing bonding strength and stress transfer efficiency, avoiding brittle cracking caused by stress concentration, and improving ductility and shear stability. Meanwhile, the hydration film covers the inner walls of pores and reduces connectivity, and the water retention effect promotes sufficient hydration of cementitious materials and increases C-S-H gel content, further densifying the matrix, reducing early cracks, and fundamentally preventing mine water intrusion.

Therefore, the multi-component synergistic modification of FA-GGBS-PPCF-HPMC enables concrete to retain excellent shear-bearing capacity and ductility in high-salinity environments, while controlling the loss of compressive strength and corrosion resistance within an engineering-acceptable range. This forms a high-performance support material suitable for deep subsea roadways, providing material support for the safe and efficient support of the Sanshandao Gold Mine, and also offering a reference for the development of support materials for similar deep mines.

## 6. Conclusions

Given the extreme conditions of severe composite erosion in the deep subsea tunnels of the Shansan Island Gold Mine, the direct use of mine water to mix ordinary shotcrete often leads to a deterioration in matrix performance and reduced durability. This makes it difficult to simultaneously meet the requirements for stress relief due to large deformations in the surrounding rock and resistance to erosion, thereby limiting the long-term safe service life of the tunnels. Additionally, there are currently no viable methods for the on-site recycling of mine water resources. This study employs a mineral admixture of fly ash and ground granulated blast furnace slag (GGBS), combined with polypropylene coarse fibers and hydroxypropyl methylcellulose (HPMC). Based on the response surface method, a multi-objective mix design optimization for mine water-mixed shotcrete was conducted, yielding the following main conclusions:

(1) A quadratic polynomial regression model constructed using compressive strength, direct shear strength, and chloride ion electrical flux at 6 h, as response variables demonstrated high significance and reliable fitting performance. The optimal mix design was determined as follows: an FA/GGBS composite admixture ratio of 3:7, a cement replacement rate of 20%, a PPCF content of 3.3%, and an HPMC content of 0.18%.

(2) Mechanical testing revealed that the optimal mixture achieved a peak compressive strength of 25.24 MPa, a slight decrease of 9.98% compared to the reference group’s 28.04 MPa, while the residual strength increased from 4.85 MPa to 8.24 MPa (a 41.14% increase); the elastic moduli before and after the peak decreased to 16.70 GPa and 7.20 GPa, respectively, and the failure mode changed from brittle splitting to ductile spalling and reticulated cracking. In the direct shear test, the peak and residual shear strengths increased by 18.1% and 51.7%, respectively, to 8.08 MPa and 3.14 MPa. The displacements before and after the peak increased, and the failure mode changed from a single through-crack to a network-like composite cracking. The mechanism involves the polypropylene coarse fibers dissipating energy through three-dimensional bridging-to dissipate energy and enhance residual load-bearing capacity; HPMC reinforces the bond at the fiber-matrix interface, further unlocking the material’s ductility, resulting in significantly enhanced ductile yielding and resistance to compressive failure, albeit at the cost of a slight reduction in peak strength.

(3) In the optimal group, the chloride ion electrical flux at 6 h decreased from 1176 C in the reference group to 778 C, a reduction of 33.8%, indicating a significant improvement in chloride ion transport barrier performance. Field industrial trials demonstrated that the modified shotcrete used for tunnel support exhibited no significant water seepage and is suitable for extreme conditions in deep-sea environments characterized by high stress and high mineralization. Replacing freshwater with mine water on-site during mixing reduces engineering water costs and provides a technical reference for the resource utilization of mine water in deep mines and the design of support systems for highly erosive tunnels.

In summary, this study introduced the response surface method into the material optimization design of shotcrete mixed with high-salinity mine water and proposed a multi-component synergistic modification approach. It achieved a synergistic improvement in mechanical load-bearing capacity, deformation and pressure relief, and resistance to chloride ion transport, providing methodological support and an engineering paradigm for durable support of deep mine tunnels using mine water as a mixing agent. It also offers a replicable technical pathway for the resource utilization of mine water and the performance enhancement of support materials in similar deep mines.

## Figures and Tables

**Table 1:** Main chemical compositions of cement and fly ash (wt%).

Components	SiO_2_	Al_2_O_3_	CaO	Fe_2_O_3_	MgO	SO_3_	Loss
Cement	20.77	5.19	62.08	3.82	3.26	2.58	2.25
Fa	45.1	24.2	5.6	0.85	2.13	2.1	4.7
GGBS	3.22	16.04	39.51		8.96	1.8	0.2

**Table 2:** Basic Properties of PPCF.

Category	Diameter	Length	Breaking Strength	Elastic Modulus	Elongation at Break
PPCF	0.8 mm	30 mm	≥500 MPa	≥3500 MPa	15–30%

**Table 3:** Ionic composition of mine water in the mining area.

Mine	Sampling Location	Cl^−^/(mg·L^−1^)	SO_4_^2−^/(mg·L^−1^)	Na^+^/(mg·L^−1^)	Ca^2+^/(mg·L^−1^)	Mg^2+^/(mg·L^−1^)
Xishan Mining Area	−1005 m	27,400	2650	14,500	1740	1016

**Table 4:** Level values of design factors.

Factors	Variables	Level Range		
		−1	0	1
HPMC (%)	$X_{1}$	0.1	0.35	0.6
PPCF (%)	$X_{2}$	3	5	7
Fa/GGBS	$X_{3}$	3/7	5/5	7/3

**Table 5:** Analysis of variance table of regression equation.

No.	X_1_ HPMC (%)	X_2_ PPCF (%)	X_3_ Fa/Fe	Mine Water (kg/m^3^)	Cement (kg/m^3^)	River Sand (kg/m^3^)	Fe (kg/m^3^)	Fa (kg/m^3^)	HPMC (kg/m^3^)	PPCF (kg/m^3^)	Compressive (MPa)	Shear Strength (MPa)	Electrical Flux (C)
1	0.1	2	1.381	200	381.333	1620	47.667	47.667	0.476	4.661	23.51	7.12	973
2	0.3	2	1.381	200	381.333	1620	47.667	47.667	1.43	4.661	23.07	6.92	963
3	0.1	7	1.381	200	381.333	1620	47.667	47.667	0.952	2.072	20.02	6.99	953
4	0.3	7	1.381	200	381.333	1620	47.667	47.667	0.952	7.25	19.63	6.79	947
5	0.1	4.5	0.429	200	381.333	1620	28.667	66.667	0.952	4.661	23.89	8.23	979
6	0.3	4.5	0.429	200	381.333	1620	28.667	66.667	0.952	4.661	23.43	7.8	963
7	0.1	4.5	2.333	200	381.333	1620	66.667	28.667	0.476	2.072	21.23	7.24	1107
8	0.3	4.5	2.333	200	381.333	1620	66.667	28.667	0.476	7.25	20.85	7.03	1094
9	0.2	2	0.429	200	381.333	1620	28.667	66.667	1.43	2.072	25.77	7.75	858
10	0.2	7	0.429	200	381.333	1620	28.667	66.667	1.43	7.25	21.93	7.61	858
11	0.2	2	2.333	200	381.333	1620	66.667	28.667	0.476	4.661	22.75	6.81	976
12	0.2	7	2.333	200	381.333	1620	66.667	28.667	0.476	4.661	19.36	6.68	958
13	0.2	4.5	1.381	200	381.333	1620	47.667	47.667	1.43	4.661	23.46	7.96	992
14	0.2	4.5	1.381	200	381.333	1620	47.667	47.667	1.43	4.661	23.58	7.98	986
15	0.2	4.5	1.381	200	381.333	1620	47.667	47.667	0.952	4.661	23.43	7.91	995
16	0.2	4.5	1.381	200	381.333	1620	47.667	47.667	0.952	4.661	23.36	7.94	994
17	0.2	4.5	1.381	200	381.333	1620	47.667	47.667	0.952	4.661	23.48	7.81	982

**Table 6:** Analysis of variance table of regression equation.

	df	Compressive			Shear Strength			Electrical Flux		
		F-Value	*p*-Value	Significance	F-Value	*p*-Value	Significance	F-Value	*p*-Value	Significance
Model	9	720.42	<0.0001	significant	123.21	<0.0001	significant	123.21	<0.0001	significant
A-HPMC	1	46.7	0.0002		35.68	0.0006		35.68	0.0006	
B-XianWei	1	3357.43	<0.0001		9.27	0.0187		9.27	0.0187	
C-Fa/Fe	1	1963.98	<0.0001		434.68	<0.0001		434.68	<0.0001	
AB	1	0.0837	0.7807		0	1		0	1	
AC	1	0.2143	0.6574		3.19	0.1171		3.19	0.1171	
BC	1	6.78	0.0352		0.0066	0.9375		0.0066	0.9375	
A^2^	1	567.99	<0.0001		100.84	<0.0001		100.84	<0.0001	
B^2^	1	457.88	<0.0001		489.54	<0.0001		489.54	<0.0001	
C^2^	1	6.64	0.0366		2.13	0.1881		2.13	0.1881	
Residual	7									
Lack of Fit	3	1.38	0.37	not significant	0.6536	0.6214	not significant	0.6536	0.6214	not significant
Pure Error	4									

Note: df represents degrees of freedom; a *p*-value less than 0.05 indicates that the model term is significant, while a *p*-value greater than 0.1000 indicates that the model term is not significant.

**Table 7:** Analysis results of model reliability test.

Group	Std. Dev.	Mean	C.V. %	$R^{2}$	Adjusted $R^{2}$	Predicted $R^{2}$	Adeq Precision
Model *Y*_1_	0.0864	22.51	0.3838	0.9989	0.9975	0.9904	94.2792
Model *Y*_2_	0.0616	7.45	0.8268	0.9937	0.9857	0.9604	32.1686
Model *Y*_3_	7.53	975.18	0.7718	0.9936	0.9854	0.9268	43.2427

**Table 8:** Experimental values and predicted values of optimization scheme.

No.	Test 1	Test 2	Test 3	Predicted Value	Error
Compressive	24.62 (MPa)	26.43 (MPa)	24.67 (MPa)	25.42 (MPa)	3.36%
Shear strength	7.89 (MPa)	8.33 (MPa)	8.02 (MPa)	8.23 (MPa)	2.63%
Electrical flux	803.61 (C)	734.79 (C)	795.11 (C)	771 (C)	4.02%

## Data Availability

The original contributions presented in this study are included in the article. Further inquiries can be directed to the corresponding author.
